# Genome-wide transcriptome study in wheat identified candidate genes related to processing quality, majority of them showing interaction (quality x development) and having temporal and spatial distributions

**DOI:** 10.1186/1471-2164-15-29

**Published:** 2014-01-16

**Authors:** Anuradha Singh, Shrikant Mantri, Monica Sharma, Ashok Chaudhury, Rakesh Tuli, Joy Roy

**Affiliations:** 1 National Agri-Food Biotechnology Institute (NABI), Department of Biotechnology (DBT), Government of India, C-127 Industrial Area Phase 8, Mohali 160 071, Punjab, India; 2 Department of Biotechnology & Nanotechnology, Guru Jambheshwar University of Science & Technology, Hisar 125 001, Haryana, India

**Keywords:** Wheat, Chapatti, Processing quality, Development, Interaction, Transcriptome, Gene expression

## Abstract

**Background:**

The cultivated bread wheat (*Triticum aestivum* L.) possesses unique flour quality, which can be processed into many end-use food products such as bread, pasta, chapatti (unleavened flat bread), biscuit, etc. The present wheat varieties require improvement in processing quality to meet the increasing demand of better quality food products. However, processing quality is very complex and controlled by many genes, which have not been completely explored. To identify the candidate genes whose expressions changed due to variation in processing quality and interaction (quality x development), genome-wide transcriptome studies were performed in two sets of diverse Indian wheat varieties differing for chapatti quality. It is also important to understand the temporal and spatial distributions of their expressions for designing tissue and growth specific functional genomics experiments.

**Results:**

Gene-specific two-way ANOVA analysis of expression of about 55 K transcripts in two diverse sets of Indian wheat varieties for chapatti quality at three seed developmental stages identified 236 differentially expressed probe sets (10-fold). Out of 236, 110 probe sets were identified for chapatti quality. Many processing quality related key genes such as glutenin and gliadins, puroindolines, grain softness protein, alpha and beta amylases, proteases, were identified, and many other candidate genes related to cellular and molecular functions were also identified. The ANOVA analysis revealed that the expression of 56 of 110 probe sets was involved in interaction (quality x development). Majority of the probe sets showed differential expression at early stage of seed development i.e. temporal expression. Meta-analysis revealed that the majority of the genes expressed in one or a few growth stages indicating spatial distribution of their expressions. The differential expressions of a few candidate genes such as pre-alpha/beta-gliadin and gamma gliadin were validated by RT-PCR. Therefore, this study identified several quality related key genes including many other genes, their interactions (quality x development) and temporal and spatial distributions.

**Conclusions:**

The candidate genes identified for processing quality and information on temporal and spatial distributions of their expressions would be useful for designing wheat improvement programs for processing quality either by changing their expression or development of single nucleotide polymorphisms (SNPs) markers.

## Background

Bread wheat (*Triticum aestivum* L.) is the most common cultivated wheat in the world. Its flour can be processed into a wide range of food products such as bread, pasta, biscuits, unleavened flat bread (chapatti), etc. The end use quality of products is mainly dependent on processing quality, which is largely determined by balance composition of biochemical molecules in seed such as seed storage proteins [1], starch [2], phenolic compounds [3], etc. There is a continuous increasing demand for good quality products both by consumers and baking industries. For improvement of processing quality it is important to understand the genome-wide expression of genes and their temporal and spatial distributions. Of several approaches for genome-wide study, microarrays comprising a large amount of probe sets of transcripts can be useful for the identification of differentially expressed genes in diverse or contrasting set of genotypes for trait of interest. In this study, two traditionally known good and two poor chapatti making Indian wheat varieties were used to identify the candidate genes whose expression changed due to chapatti quality using wheat microarrays.

Transcriptome analysis has been used to improve genome-wide understanding of molecular mechanism of gene expression. In wheat, it has been done using either the 8K wheat microarray chips [4,5] or 61K wheat microarray chips [5,6]. Recently, the next generation sequencing has become an important technological platform for investigating genome-wide transcriptional regulation of metabolic pathways [7]. However, in a polyploid crop such as bread wheat, sequence assembly and annotation are very challenging due to occurrence of multiple copies of gene sequences (homoeologous or paralogous genes). A reference bread wheat genome sequence, and cost-effective and faster high throughput computation system is required for making advances in wheat genomics. Despite several complications associated with microarray experiments [8], it still provides a faster and cost-effective method for genome-wide transcriptome study than the next generation sequencing approach.

Wheat microarrays have been successfully used for the identification of potential candidate genes under a wide range of biotic and abiotic stresses. For example, it has been used for studying the change in transcriptome patterns under biotic stresses such as Fusarium head blight [9-11], leaf rust [12], and insect attack [13]. Similarly, it has also been used for abiotic stress conditions such as cold [14], drought tolerance [15], high nitrogen level [16], etc. Change in transcriptional profiling during seed development was studied using wheat microarrays [4]. It has been also used to study metabolic biosynthesis pathway such as starch [5] and reactive oxygen species (ROS) [17]. Wheat microarrays have been used to screen and identify seed-specific genes using digital differential display (DDD) tools [18]. The translocation breakpoints were mapped using wheat microarrays [19]. The application of microarrays for gene expression analysis has been discussed in detail elsewhere [20].

The gene expression analysis through seed developmental stages is important for understanding temporal distribution of gene expression, as the seed matures. Three stages of major transitions in gene expression have been reported [4,6]. The first major transition is within 10 days after anthesis (DAA) where extensive cell division, expansion and differentiation occur to make milky endosperm [6,21]. The second major transition occurs at 14 DAA where starch and seed storage proteins accumulate within cells to make semi-solid endosperm [6]. The third major transition starts at 28 DAA where deposition of storage reserve decreases [6] followed by physiological maturity to turn caryopsis brown. However, the duration of the above stages may change depending upon environmental conditions during seed development. For example, Laudencia-Chingcuanco *et al.*[4] observed three stages of gene expression patterns during seed development i.e. 3 to 7 DAA (multi-cellular tissue forming structure), 7 to 14 DAA (grain filling stage), and 21 to 28 DAA (maturing and desiccation stage). Similarly, hierarchical clustering of transcriptome data from 6 to 42 DAA revealed three major groups of expressed genes i.e. 6 to 10 DAA, 12 to 21 DAA, and 28 to 42 DAA [6]. Therefore, genome-wide transcriptome study in at least three seed developmental stages is important for the identification of candidate genes whose expression changed during seed development for the improvement of processing quality.

Several statistical approaches are useful for the analysis of gene expression data from microarrays [22]. Among them, two-way ANOVA (analysis of variance) is the most common analysis method where two categorical explanatory variables of an experiment can be simultaneously analysed by any combination of one level of one variable and one level of the other variable. In this analysis, variation in the expression of a gene which is affected by the level of other gene (s) (i.e. interaction) can be determined [14]. In this study gene-specific two-way ANOVA was implemented to identify candidate genes whose expression changed due to processing quality (chapatti in this study), seed development, and interaction (quality x seed development).

Two traditionally known good chapatti making varieties, C306 [23] and Lok1 were used to identify differentially expressed genes in comparison to two varieties, Sonalika [23] and WH291, poor chapatti making varieties using wheat microarrays. The genes involved in seed development and interaction (quality x development) were also studied. Since the four varieties might differ for other traits, the involvement of the candidate genes with other traits cannot be rule out in this study. Several processing quality related key genes such as seed storage protein genes (gliadins and glutenins), which were known to effect processing quality of several end-use products such as bread, biscuit, noodles, etc., shown very high difference in their expression between good and poor chapatti making varieties at early stage of seed development (7 DAA). In addition, many novel candidate genes were identified between the two groups. It is evident that majority of the reported genes involved in processing quality were also involved in interaction of quality and seed development. This information would be helpful to design experiments for gene regulation at specific development stage and for the identification of variations within genes such as single nucleotide polymorphisms (SNPs) useful for the improvement of processing quality in wheat through molecular breeding.

## Results and discussion

Genome-wide transcriptome analysis was done between good and poor quality varieties at three seed developmental stages (7, 14, and 28 DAA) to identify the candidate genes whose expressions changed due to quality, development, and interaction (quality x development). A total of 60,130 probe sets (out of 61,290) qualified gene expression quality criterion where at least 100% of samples in any one out of 6 conditions (discussed later in Methods) had values between 20.0 and 100.0th percentile in the normalized data. Gene specific two-way ANOVA analysis with multiple test corrections (Benjamini-Hochberg’s FDR) of the 60,130 probe sets identified 35,472 probe sets (about 59%), which satisfied the corrected *p* value of 0.05 (see Additional file 1). A similar number of the expressed genes (31,768) were identified by Laudencia-Chingcuanco *et al.*[14] among wheat genotypes during cold acclimation and vernalization using gene-specific ANOVA. The analysis partitioned the variation in expression of the probe sets into quality (3,126 probe sets), development (34,604 probe sets), interaction (quality x development) (1,732 probe sets), and expected by chance i.e. errors (156 probe sets) (see Additional file 2, Table 1). The number of probe sets passed the test in this study is higher than the number reported by Wan *et al.*[6] who had identified 14,550 probe sets showing significant differential expression through development in a single wheat variety. In this study, a large number of genes were involved in quality and seed development referring to the involvement of many pathways and cellular processes.

**Table 1:** Summary of gene specific two-way ANOVA analysis of the expression data of the 35,472 probe sets

**Source of variation**	**Number of probe sets whose expression varied at the corrected *p *** **value of *P*** **< 0.05**
Quality	3,126
Seed development	34,604
Quality x seed development	1,732
Expected by chance i.e. error	156

Fold change analysis of the expression data of the 35,472 probe sets identified 236 probe sets, whose expressions were at least 10-fold between good and poor quality varieties in at least one of the three sources of variations [quality, development, and interaction (quality x development)] (Table 2). Among them, 110, 219, and 85 probe sets were involved in quality, development, and interaction (quality x development), respectively (Table 2, see Additional file 3).

**Table 2:** Detail of fold change analysis (at least 10-fold) of the expression data of the 35,472 probe sets between good and poor quality varieties

**Source of variation**	**Quality**		**Seed development**		**Quality x seed development**	
Number of probe sets (at least 10-fold)	110		219		85	
Regulation (up/down)	Up	Down	Up	Down	Up	Down
Seed developmental stage						
7 DAA	58	19	155	24	53	16
14 DAA	5	6	7	2	3	1
28 DAA	27	11	33	10	11	3

The probe sets were identified through gene specific two-way ANOVA analysis.

### Identification of candidate genes whose expression changed due to quality

Total of 110 probe sets were identified for quality which showed at least ten-fold differential expression between good and poor quality varieties in at least one of the three condition pairs (‘Good-7 DAA’ vs ‘Poor-7 DAA’, ‘Good-14 DAA’ vs ‘Poor-14 DAA’, and ‘Good-28 DAA’ vs ‘Poor-28 DAA’) (Tables 2 and 3, see Additional file 3, Figures 1 and 2A). Using blastx similarity search of the sequence of the 110 probe sets at NCBI, the putative gene function was assigned to 67 (about 61% = 67/110), hypothetical proteins to 37 (33.6% = 37/110) probe sets, and the function was not assigned to the remaining 6 probe sets. Out of 110, 67, 1, and 28 probe sets were differentially expressed at 7, 14, and 28 DAA, respectively and the remaining 14 probe sets were differentially expressed in either two or three seed developmental stages (Figure 2A).

**Table 3:** Detail of the 110 probe sets identified for processing quality showing at least 10-fold differential expression between good and poor quality wheat varieties in at least one of the three seed developmental stages i.e. 7, 14, and 28 days after anthesis (DAA)

**Probe set ID**	**Putative gene function**	**7 DAA**		**14 DAA**		**28 DAA**		**Quality**	**Seed development**	**Quality x seed development**
		**Fold change**	**Regulation**	**Fold change**	**Regulation**	**Fold change**	**Regulation**			
Ta.1345.2.S1_x_at	14 kDa proline-rich protein DC2.15 [*Triticum urartu*]	12.1	Down	1.1	Down	1.0	Up	+	+	+
Ta.1345.1.S1_x_at	14 kDa proline-rich protein DC2.15 [*Triticum urartu*]	11.1	Down	1.0	Up	1.1	Down	+	+	+
TaAffx.128648.2.A1_at	2′-deoxymugineic-acid 2′-dioxygenase [*Triticum urartu*]	15.3	Up	4.3	Up	1.3	Up	+	+	-
Ta.24114.10.S1_x_at	Alpha/beta-gliadin [*Triticum aestivum*]	54.1	Up	1.1	Up	1.1	Up	+	+	+
Ta.10028.1.S1_at	Alpha-amylase/subtilisin inhibitor [*Triticum urartu*]	30.9	Up	5.3	Up	1.1	Up	+	+	-
Ta.27778.4.S1_x_at	Alpha-gliadin [*Triticum aestivum*]	284.7	Up	1.3	Up	1.2	Down	+	+	+
Ta.15268.1.S1_x_at	Alpha-gliadin [*Triticum aestivum*]	110.3	Up	1.1	Up	1.1	Up	+	+	+
Ta.27778.2.S1_x_at	Alpha-gliadin [*Triticum aestivum*]	209.5	Up	1.5	Up	1.8	Down	+	+	+
Ta.24114.1.S1_x_at	Alpha-gliadin protein [*Triticum dicoccoides*]	171.4	Up	1.0	Up	1.2	Down	+	+	+
Ta.21787.1.S1_at	Aspartic proteinase nepenthesin-1 [*Triticum urartu*]	12.5	Down	1.0	Down	1.1	Up	+	+	+
Ta.27777.1.S2_s_at	Avenin-like b1	122.6	Up	1.1	Up	1.0	Up	+	+	+
Ta.27777.1.S2_at	Avenin-like b1	57.6	Up	1.0	Up	1.3	Up	+	+	+
Ta.27712.1.S1_at	Avenin-like protein [*Triticum aestivum*]	86.6	Up	1.1	Up	1.3	Up	+	+	+
Ta.9799.1.S1_at	Avenin-like protein [*Triticum aestivum*]	45.0	Up	2.7	Up	2.9	Up	+	+	-
Ta.27780.1.S1_x_at	Beta-amylase [*Aegilops tauschii*]	58.6	Up	1.2	Down	1.1	Down	+	+	+
Ta.27780.2.A1_a_at	Beta-amylase [*Triticum urartu*]	156.1	Up	1.0	Down	1.2	Down	+	+	+
Ta.27780.2.A1_x_at	Beta-amylase [*Triticum urartu*]	122.4	Up	1.0	Up	1.2	Down	+	+	+
Ta.27780.3.S1_x_at	Beta-amylase, partial [*Triticum aestivum*]	72.0	Up	1.0	Down	1.1	Up	+	+	+
Ta.9809.2.S1_at	Beta-fructofuranosidase, isoenzyme 4 [*Aegilops tauschii*]	12.6	Up	1.7	Up	1.1	Up	+	+	-
Ta.27445.1.S1_at	Cell division protease ftsH-like protein, chloroplastic [*Aegilops tauschii*]	9.2	Down	13.4	Down	28.6	Down	+	-	-
Ta.6984.1.A1_at	Chromodomain-helicase-DNA-binding protein 4 [*Triticum urartu*]	58.0	Down	7.0	Down	16.9	Down	+	+	-
Ta.14507.2.S1_at	Cytosolic Fe-S cluster assembly factor NUBP1-like protein [*Triticum urartu*]	29.9	Down	3.6	Down	7.6	Down	+	-	-
TaAffx.108987.1.S1_at	DNA-directed RNA polymerase subunit beta [*Triticum urartu*]	2.0	Down	5.7	Up	15.2	Up	+	+	+
Ta.30782.4.S1_at	Gamma gliadin [*Triticum aestivum*]	98.0	Up	1.0	Up	1.0	Up	+	+	+
Ta.28792.1.S1_x_at	Gamma-gliadin [*Triticum aestivum*]	146.9	Up	1.0	Up	2.2	Up	+	+	+
Ta.24114.8.S1_x_at	Gamma-gliadin [*Triticum aestivum*]	194.8	Up	1.1	Up	1.8	Up	+	+	+
Ta.6175.1.S1_at	Gamma-gliadin [*Triticum aestivum*]	105.9	Up	1.2	Up	1.5	Down	+	+	+
Ta.6175.1.S1_x_at	Gamma-gliadin [*Triticum aestivum*]	80.9	Up	1.4	Up	1.2	Down	+	+	+
Ta.30782.2.S1_x_at	Gamma-gliadin *[Triticum aestivum*]	51.3	Up	1.2	Up	1.3	Up	+	+	+
Ta.24114.8.S1_at	Gamma-gliadin [*Triticum aestivum*]	168.9	Up	1.0	Up	1.9	Up	+	+	+
Ta.30782.2.S1_a_at	Gamma-gliadin [*Triticum aestivum*]	28.2	Up	1.2	Up	1.5	Up	+	+	-
Ta.160.3.S1_x_at	Gamma-gliadin, partial [*Triticum aestivum*]	13.6	Up	3.6	Up	3.5	Up	+	+	-
Ta.160.2.S1_x_at	Gamma-gliadin, partial [*Triticum aestivum*]	13.3	Up	3.8	Up	3.5	Up	+	+	-
Ta.160.1.S1_x_at	Gamma-gliadin, partial [*Triticum aestivum*]	14.6	Up	1.8	Up	2.7	Up	+	+	-
Ta.7430.1.S1_at	GATA transcription factor 17 [*Triticum urartu*]	11.4	Up	5.3	Up	6.4	Up	+	-	-
TaAffx.27375.1.S1_at	General transcription factor IIF subunit 2 [*Triticum urartu*]	2.4	Down	5.1	Up	11.4	Up	+	+	+
Ta.23142.11.S1_x_at	Gliadin/avenin-like seed protein [*Triticum aestivum*]	124.1	Up	1.1	Up	1.1	Down	+	+	+
Ta.23142.4.S1_s_at	Gliadin/avenin-like seed protein [*Triticum aestivum*]	175.9	Up	1.0	Up	1.0	Up	+	+	+
Ta.2415.2.S1_a_at	Gliadin/avenin-like seed protein [*Triticum aestivum*]	17.4	Up	2.1	Up	1.1	Down	+	-	-
Ta.24114.7.A1_at	Granule-bound starch synthase I [*Triticum aestivum*]	6.7	Down	26.9	Down	92.8	Down	+	+	-
Ta.5129.2.A1_a_at	Heat stress transcription factor A-9 [*Aegilops tauschii*]	7.5	Down	5.4	Down	25.1	Down	+	+	-
Ta.24963.1.S1_x_at	HMW-glutenin subunit 1By9, truncated [*Triticum aestivum subsp. tibeticum*]	60.4	Up	1.1	Down	1.1	Up	+	+	+
Ta.24298.1.S1_x_at	HMW-glutenin subunit Dx5 [*Triticum aestivum*]	56.7	Up	1.1	Down	1.0	Up	+	+	+
Ta.25839.1.A1_a_at	Hypothetical protein F775_04832 [*Aegilops tauschii*]	26.6	Up	4.6	Up	24.9	Up	+	+	-
Ta.7509.3.S1_at	Hypothetical protein F775_05928 [*Aegilops tauschii*]	14.0	Down	26.9	Down	22.0	Down	+	-	-
Ta.24550.1.S1_s_at	Hypothetical protein F775_05934 [*Aegilops tauschii*]	16.7	Up	1.4	Down	1.2	Down	+	+	+
Ta.28759.1.A1_at	Hypothetical protein F775_09062 [*Aegilops tauschii*]	5.7	Up	2.7	Up	14.7	Up	+	-	-
Ta.19222.1.S1_at	Hypothetical protein F775_11525 [*Aegilops tauschii*]	29.8	Up	6.9	Up	5.3	Up	+	+	-
Ta.28744.1.S1_at	Hypothetical protein F775_23873 [*Aegilops tauschii*]	11.3	Up	11.0	Up	35.2	Up	+	+	-
Ta.23681.1.S1_a_at	Hypothetical protein F775_24401 [*Aegilops tauschii*]	13.9	Up	8.6	Up	19.3	Up	+	+	-
Ta.9888.1.A1_at	Hypothetical protein F775_26991 *[Aegilops tauschii*]	12.7	Up	1.1	Up	1.1	Up	+	+	+
Ta.8883.2.S1_at	Hypothetical protein F775_27368 [*Aegilops tauschii*]	12.0	Down	7.9	Down	9.2	Down	+	-	-
Ta.14050.1.S1_at	Hypothetical protein F775_27373 [*Aegilops tauschii*]	2.8	Up	3.7	Up	37.3	Up	+	-	-
Ta.22853.1.S1_at	Hypothetical protein F775_27669 [*Aegilops tauschii*]	10.1	Down	2.8	Down	1.1	Up	+	+	+
Ta.12328.2.A1_at	Hypothetical protein F775_32343 [*Aegilops tauschii*]	1.1	Down	2.3	Up	10.1	Up	+	+	-
Ta.8621.1.S1_at	Hypothetical protein F775_43701 [*Aegilops tauschii*]	10.8	Down	1.2	Up	1.1	Up	+	+	+
Ta.24736.1.S1_at	Hypothetical protein F775_43830 [*Aegilops tauschii*]	550.2	Up	31.2	Up	8.0	Up	+	+	-
Ta.24218.1.S1_at	Hypothetical protein TRIUR3_02605 [*Triticum urartu*]	11.6	Up	6.9	Up	3.6	Up	+	-	-
Ta.5278.1.S1_at	Hypothetical protein TRIUR3_04715 [*Triticum urartu*]	10.1	Down	4.2	Down	3.0	Down	+	-	-
Ta.14446.1.A1_at	Hypothetical protein TRIUR3_05569 [*Triticum urartu*]	14.8	Down	13.2	Down	9.9	Down	+	-	-
Ta.5243.2.S1_a_at	Hypothetical protein TRIUR3_06223 [*Triticum urartu*]	2.3	Down	4.9	Down	19.0	Down	+	+	-
Ta.2025.1.S1_at	Hypothetical protein TRIUR3_06809 [*Triticum urartu*]	1.6	Down	86.3	Down	1727.5	Down	+	+	+
Ta.23013.3.S1_s_at	Hypothetical protein TRIUR3_09069 [*Triticum urartu*]	31.5	Down	1.4	Down	1.4	Down	+	+	+
Ta.6018.1.S1_x_at	Hypothetical protein TRIUR3_12951 [*Triticum urartu*]	10.7	Up	1.1	Up	1.0	Up	+	+	+
Ta.24550.2.S1_s_at	Hypothetical protein TRIUR3_13108 [*Triticum urartu*]	28.6	Up	1.4	Down	1.2	Down	+	+	+
Ta.24550.2.S1_at	Hypothetical protein TRIUR3_13108 [*Triticum urartu*]	21.1	Up	1.5	Down	1.1	Down	+	+	+
TaAffx.80038.1.S1_at	Hypothetical protein TRIUR3_13205 [*Triticum urartu*]	11.9	Down	1.4	Up	1.1	Up	+	+	+
TaAffx.82110.1.S1_at	Hypothetical protein TRIUR3_18607 [*Triticum urartu*]	1.4	Down	2.5	Up	10.5	Up	+	+	-
Ta.7158.1.S1_at	Hypothetical protein TRIUR3_24125 [*Triticum urartu*]	108.1	Up	6.8	Up	4.5	Up	+	+	-
Ta.11896.1.A1_s_at	Hypothetical protein TRIUR3_24659 [*Triticum urartu*]	24.9	Up	7.9	Up	3.7	Up	+	-	-
TaAffx.80118.1.S1_at	Hypothetical protein TRIUR3_25902 [*Triticum urartu*]	20.1	Down	5.8	Down	32.6	Down	+	+	-
Ta.4497.1.S1_at	Hypothetical protein TRIUR3_27348 [*Triticum urartu*]	10.3	Down	1.2	Up	1.1	Up	+	+	+
TaAffx.5865.2.A1_at	Hypothetical protein TRIUR3_27593 [*Triticum urartu*]	1.4	Down	2.9	Up	14.2	Up	+	+	-
TaAffx.78552.1.S1_at	Hypothetical protein TRIUR3_27901 [*Triticum urartu*]	14.7	Up	1.9	Up	3.4	Up	+	+	+
TaAffx.78552.1.S1_x_at	Hypothetical protein TRIUR3_27901 [*Triticum urartu*]	11.7	Up	1.7	Up	2.9	Up	+	-	-
Ta.5785.1.S1_at	Hypothetical protein TRIUR3_27988 [*Triticum urartu*]	1.1	Up	1.1	Up	14.3	Up	+	+	+
TaAffx.5496.1.S1_at	Hypothetical protein TRIUR3_29836 [*Triticum urartu*]	16.7	Up	10.6	Up	2.9	Up	+	+	-
Ta.21106.1.A1_at	Hypothetical protein TRIUR3_30277 [*Triticum urartu*]	4.9	Up	5.7	Up	16.2	Up	+	+	-
Ta.14723.1.S1_at	Hypothetical protein TRIUR3_33733 [*Triticum urartu*]	1.7	Down	3.5	Up	11.3	Up	+	+	+
Ta.3527.1.S1_at	Hypothetical protein TRIUR3_34067 [*Triticum urartu*]	6.2	Up	8.1	Up	28.3	Up	+	+	-
TaAffx.30606.1.S1_at	Katanin p80 WD40 repeat-containing subunit B1-like Protein 1 [*Triticum urartu*]	1.2	Down	2.3	Up	14.0	Up	+	+	-
Ta.23142.5.S1_x_at	LMW-glutenin [*Triticum aestivum*]	57.8	Up	1.0	Up	1.3	Up	+	+	+
Ta.14625.1.S1_x_at	LMW-glutenin [*Triticum aestivum*]	106.0	Up	1.1	Down	1.2	Down	+	+	+
Ta.131.1.S1_at	LMW-glutenin subunit, partial [*Triticum aestivum*]	58.9	Up	1.1	Up	1.2	Up	+	+	+
TaAffx.109582.1.S1_s_at	LRR receptor-like serine/threonine-protein kinase FLS2 [*Triticum urartu*]	3.7	Down	9.2	Up	10.3	Up	+	+	+
Ta.12643.5.S1_x_at	No significant similarity found	92.5	Up	1.2	Up	1.0	Up	+	+	+
Ta.7825.1.S1_s_at	No significant similarity found	1.0	Up	13.5	Up	1.2	Up	+	+	+
TaAffx.104444.1.S1_at	No significant similarity found	47.5	Down	5.4	Down	4.4	Down	+	+	-
TaAffx.31445.1.S1_at	No significant similarity found	1.4	Down	3.6	Up	11.1	Up	+	+	-
Ta.19222.1.S1_x_at	No significant similarity found	14.1	Up	3.9	Up	3.2	Up	+	-	-
TaAffx.91902.1.A1_at	No significant similarity found	4.2	Up	5.5	Up	32.6	Up	+	-	-
Ta.23896.1.S1_at	Omega gliadin [*Triticum aestivum*]	33.1	Up	1.4	Up	1.5	Up	+	+	-
Ta.23366.2.S1_at	Peroxidase 66 [*Triticum urartu*]	2.6	Up	10.0	Up	60.0	Up	+	+	-
Ta.23366.2.S1_x_at	Peroxidase 66 [*Triticum urartu*]	2.4	Up	5.6	Up	28.9	Up	+	+	-
Ta.4957.1.S1_at	Peroxisomal acyl-coenzyme A oxidase 1 [*Triticum urartu*]	26.6	Down	3.6	Down	7.9	Down	+	+	-
Ta.12764.1.A1_at	Peroxisomal multifunctional enzyme type 2 [*Aegilops tauschii*]	1.5	Down	1.3	Down	14.5	Down	+	+	+
Ta.14489.1.S1_at	pTACR7 [*Triticum aestivum*]	2.4	Down	7.6	Down	29.0	Down	+	+	-
TaAffx.118782.1.A1_at	Putative disease resistance protein RGA4 [*Triticum urartu*]	2.8	Down	1.7	Down	10.7	Down	+	+	+
Ta.23352.1.S1_at	Putative serine/threonine-protein kinase-like protein CCR3 [*Aegilops tauschii*]	10.8	Up	5.8	Up	6.1	Up	+	-	-
TaAffx.112770.1.S1_x_at	Retrovirus-related Pol polyprotein from transposon TNT 1-94 [*Triticum urartu*]	2.6	Down	6.4	Up	12.3	Up	+	+	+
TaAffx.112770.1.S1_at	Retrovirus-related Pol polyprotein from transposon TNT 1-94 [*Triticum urartu*]	1.7	Down	5.4	Up	10.7	Up	+	+	+
TaAffx.27343.2.S1_at	Serine/threonine-protein phosphatase 7 long form-like Protein [*Triticum urartu*]	1.3	Down	2.1	Up	11.5	Up	+	+	+
Ta.2434.3.A1_at	S-formylglutathione hydrolase [*Aegilops tauschii*]	13.7	Down	11.9	Down	9.6	Down	+	-	-
Ta.1622.2.S1_at	Sugar transporter ERD6-like protein 5 [*Aegilops tauschii*]	10.0	Down	2.4	Down	1.4	Down	+	+	-
TaAffx.109268.1.S1_x_at	TaCBF11 [*Triticum aestivum*]	1.2	Down	2.1	Up	10.0	Up	+	+	+
Ta.10170.1.S1_at	Trypsin inhibitor CMc [*Aegilops tauschii*]	32.9	Up	1.2	Up	1.7	Up	+	+	-
Ta.13439.1.S1_a_at	Trypsin inhibitor-Bowman-Birk type	84.5	Up	2.1	Up	3.0	Up	+	+	-
TaAffx.6814.1.S1_at	Wall-associated receptor kinase 5 [*Aegilops tauschii*]	1.2	Down	1.9	Up	11.0	Up	+	+	+
TaAffx.111195.1.S1_at	Zf-MYND domain-containing protein [*Triticum aestivum*]	1.7	Down	3.5	Up	13.3	Up	+	+	+
TaAffx.86295.1.S1_at	Zf-MYND domain-containing protein [*Triticum aestivum*]	1.3	Down	2.1	Up	10.4	Up	+	+	-

The majority of the probe sets also involved in seed development stage and interaction (quality x development) as identified through gene-specific two-way ANOVA analysis. The function to the probe sets was assigned through blastx at NCBI website.

+ = involved, - = not involved.

**Figure 1:**
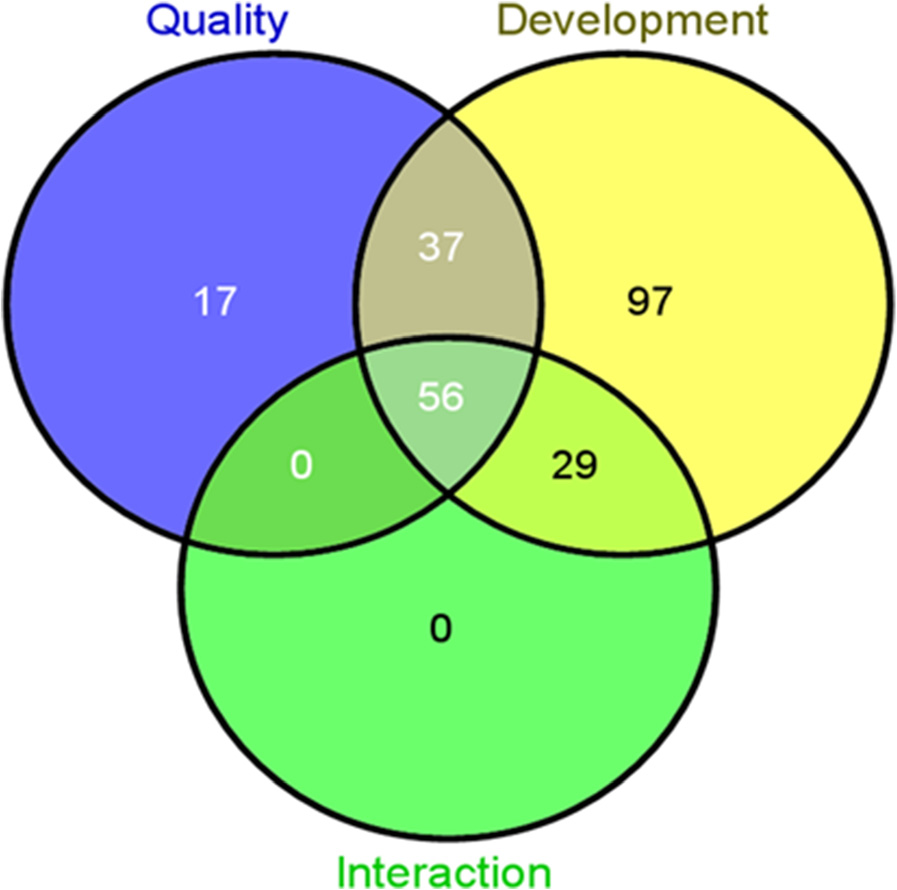
**Venn diagrams showing the number of probe sets of the candidate genes identified for quality, seed development, and interaction (quality x seed development).** The probe sets were identified by gene specific two-way ANOVA showing at least 10-fold differential expression between two good (C306 and Lok1) and poor (Sonalika and WH291) chapatti making varieties.

**Figure 2:**
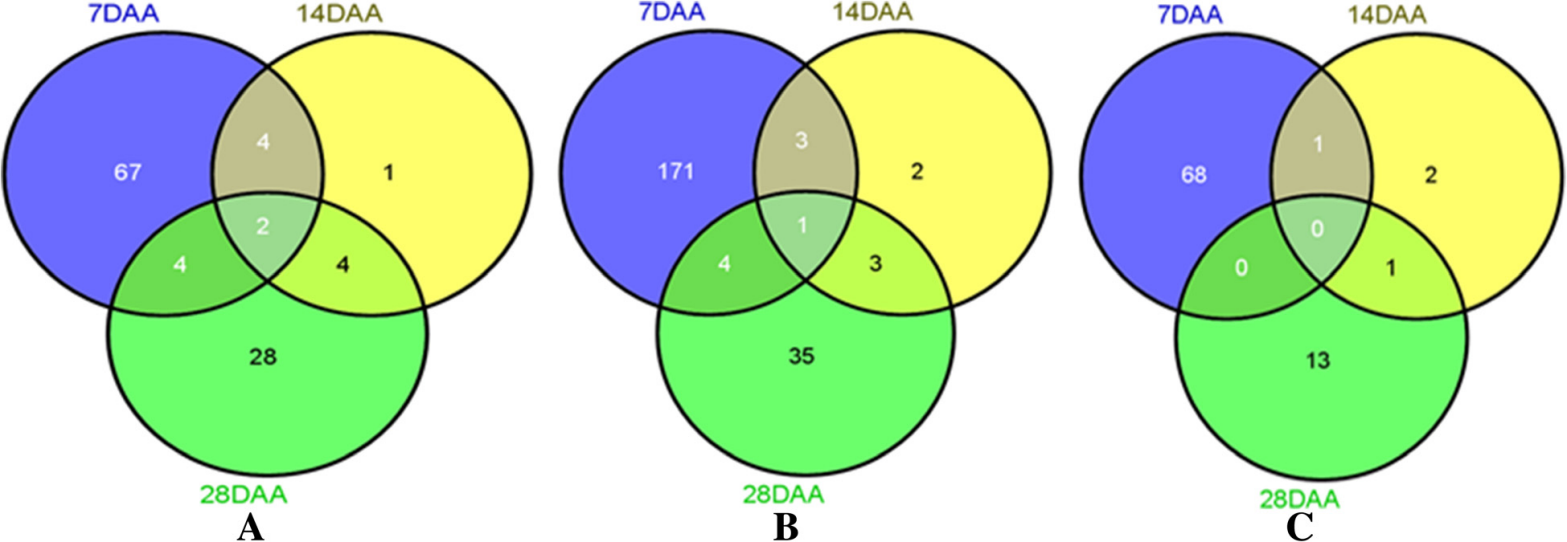
**Venn diagrams showing the number of probe sets identified in three seed developmental stages i.e. 7, 14, and 28 days after anthesis (DAA) for (A) quality, (B) seed development, and (C) interaction (quality x seed development). (A)**: out of 110 probe sets involved in quality, 67, 1 and 28 probe sets were differentially expressed at 7, 14, and 28 DAA, respectively; **(B)**: out of 219 probe sets involved in seed development, 171, 2, and 35 probe sets were differentially expressed at 7, 14, and 28 DAA, respectively; and **(C)**: out of 85 probe sets involved in interaction of quality and development, 68, 2, and 13 probe sets were differentially expressed at 7, 14, and 28 DAA, respectively.

Out of the 67, 29 probe sets (43% = 29/67), were mainly related to seed storage protein genes (Table 3). These genes were gliadin (alpha-/beta-, gamma, and omega gliadins), glutenin (low molecular weight and high molecular weight glutenin subunits), and avenin. Avenin is oat counterpart to gliadin. Glutenin and gliadin proteins make gluten matrix in dough which determines visco-elastic properties of wheat dough during processing. They mainly determine the processing quality of wheat end-use products [1,24]. High molecular weight glutenin subunit (HMW-GS) contributes mainly to gluten elasticity property and gliadins to its viscosity property [25]. HMW-GS was strongly correlated with bread making quality of wheat [26]. Gliadin contributes to gluten functional property [27-31]. Xu *et al.*[32] suggested that gliadin plays an important role in adjusting and controlling gluten’s viscoelastic properties rather than only just a diluent of gluten’s functional properties. Interestingly, at later stages of seed development (i.e. 14 and 28 DAA), these probe sets did not show much difference in expression between good and poor quality varieties.

The differential expression of the 29 probe sets of seed storage protein genes, key genes for processing quality, were analysed in pairwise among the four varieties to study variation in expression level among them (Table 4, see Additional file 4). It indicates that WH291 is more diverse than Sonalika with that of C306 and Lok1 at expression level of seed storage protein genes. Therefore, among them, the poor chapatti making quality, WH291 can be used with either of two good chapatti making varieties, C306 or Lok1 for molecular breeding.

**Table 4:** Pairwise comparisons of differential expression of the probe sets related to seed storage protein genes at early stage of seed development i.e. 7 days after anthesis (DAA) among the four Indian wheat varieties, viz. C306 and Lok1 were good and Sonalika and WH291 were poor chapatti making varieties

**Probe set ID**	**Function**	**Good vs poor**	**C306 vs Sonalika**	**C306 vs WH291**	**Lok1 vs Sonalika**	**Lok1 vs WH291**	**C306 vs Lok1**	**Sonalika vs WH291**
Ta.24298.1.S1_x_at	HMW-glutenin subunit Dx5	56.7	11.0	104.8	29.6	288.6	-2.8	10.6
Ta.24963.1.S1_x_at	HMW-glutenin subunit 1By9	60.4	13.5	70.0	54.2	303.0	-3.9	4.8
Ta.131.1.S1_at	LMW-glutenin	58.9	19.7	76.7	47.2	175.0	-2.3	3.7
Ta.14625.1.S1_x_at	LMW-glutenin	106.0	39.9	121.0	90.9	313.5	-2.4	3.6
Ta.23142.5.S1_x_at	LMW-glutenin	57.8	12.3	203.0	16.6	244.2	-1.3	15.4
Ta.24114.1.S1_x_at	Alpha-gliadin	171.4	48.9	229.2	123.4	606.1	-2.6	4.4
Ta.24114.10.S1_x_at	Alpha/beta-gliadin	54.1	10.9	75.6	42.9	266.0	-4.1	6.4
Ta.27778.4.S1_x_at	Alpha-gliadin	284.7	100.7	320.4	277.7	830.2	-2.6	3.3
Ta.27778.2.S1_x_at	Alpha-gliadin	209.5	66.7	215.8	220.4	619.9	-3.3	3.2
Ta.15268.1.S1_x_at	Alpha-gliadin	110.3	39.5	175.9	69.7	319.7	-1.8	4.3
Ta.23896.1.S1_at	Omega gliadin	33.1	25.9	91.3	14.3	40.7	1.8	3.4
Ta.160.1.S1_x_at	Gamma-gliadin	14.6	20.0	21.1	9.1	11.5	1.9	1.2
Ta.160.2.S1_x_at	Gamma-gliadin	13.3	26.0	27.0	5.9	7.4	4.3	1.2
Ta.160.3.S1_x_at	Gamma-gliadin	13.6	25.7	30.6	9.5	6.0	3.5	-1.2
Ta.28792.1.S1_x_at	Gamma-gliadin	146.9	38.2	155.4	135.1	556.7	-3.5	4.4
Ta.24114.8.S1_at	Gamma-gliadin	168.9	43.7	262.7	108.0	653.5	-2.6	6.2
Ta.24114.8.S1_x_at	Gamma-gliadin	194.8	46.6	338.5	112.0	819.7	-2.4	7.4
Ta.30782.2.S1_a_at	Gamma-gliadin	28.2	13.3	37.6	21.4	60.5	-1.6	2.8
Ta.30782.2.S1_x_at	Gamma-gliadin	51.3	28.4	80.0	35.7	77.8	-1.3	2.7
Ta.30782.4.S1_at	Gamma-gliadin	98.0	24.3	186.7	53.4	415.8	-2.1	8.0
Ta.6175.1.S1_at	Gamma-gliadin	105.9	36.7	49.1	116.2	206.2	-3.3	2.7
Ta.6175.1.S1_x_at	Gamma-gliadin	80.9	28.7	58.8	95.9	238.9	-3.6	3.0
Ta.27712.1.S1_at	Avenin	86.6	26.1	156.1	50.4	297.2	-1.8	6.3
Ta.27777.1.S2_at	Avenin	57.6	18.6	51.2	57.0	158.9	-2.3	3.0
Ta.27777.1.S2_s_at	Avenin	122.6	37.7	208.4	74.7	411.7	-1.9	5.6
Ta.9799.1.S1_at	Avenin	45.0	22.0	30.3	70.4	86.5	-2.8	1.2
Ta.23142.4.S1_s_at	Gliadin/avenin	175.9	41.2	179.3	196.0	715.6	-3.8	4.0
Ta.23142.11.S1_x_at	Gliadin/avenin	124.1	39.1	98.9	143.7	417.7	-3.9	2.8
Ta.2415.2.S1_a_at	Gliadin/avenin	17.4	14.8	46.1	7.4	18.8	1.9	2.5

The remaining probe sets showed similarity to transcription factors (GATA, IIA, heat stress TF A9), peroxidase, trypsin inhibitor, proteinase, amylases (alpha- and beta-amylases), kinases and phosphatase (serine/threonine protein kinase and phosphatase), etc. High differential expression of peroxidase, proteinase, and amylases in good chapatti varieties may have effect on processing quality. These enzymes are being extensively used in baking and food industries for making good quality end-use products.

**Figure 3:**
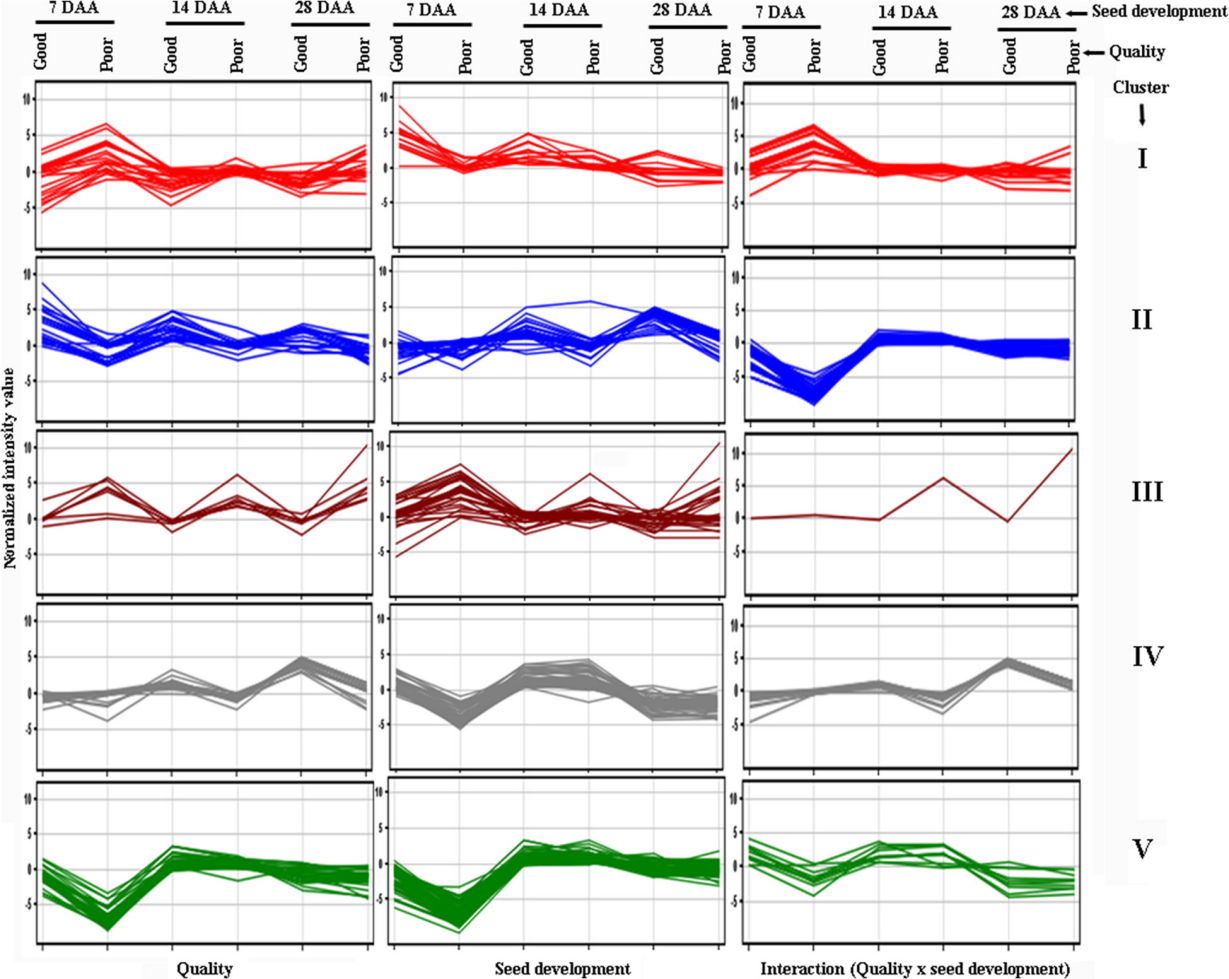
**Clustering of the expression of probe sets identified for quality, seed development, and interaction (quality x seed development) into five clusters (I, II, III, IV, and V) in three seed developmental stages, namely, 7, 14, and 28 days after anthesis (DAA).** The cluster analysis was done to identify co-expressed genes using GeneSpring software (Agilent Tech, Santa Clara, USA).

Cluster analysis of the 110 probe sets into 5 groups identified the co-expressed genes having similar expression patterns in good and poor varieties in three seed developmental stages (see Additional file 5, Figure 3). In cluster I, the probe sets showed down-regulation in good quality varieties at early stage of seed development (i.e. 7 DAA) and did not show any change at later stage of seed development. In cluster II, the probe sets showed up-regulation in good quality varieties at early stage of seed development (i.e. 7 DAA) and did not show any change at later stage of seed development. In cluster III, five probe sets showed down-regulation in good quality varieties in all three seed developmental stages. In cluster IV, the probe sets showed up-regulation in good quality varieties at later stage of seed development (28 DAA). In cluster V, the majority of the probe sets showed up-regulation in good quality varieties at early stage of seed development. Out of 40, 28 probe sets in cluster V were related to seed storage protein genes and others to beta-amylase and hypothetical proteins. One probe set for seed storage protein gene (Ta.2415.2.S1_a_at for gliadin/avenin seed protein) was clustered separately in cluster II. Cluster analysis distinctly grouped key genes for processing quality in cluster V indicating their similar expression patterns in good quality varieties i.e. up-regulated at early stage of seed development (Figure 3). The role of the remaining four clusters cannot be validated to processing quality in the present study, but they represent potential candidate genes for marker development through association studies.

### Identification of candidate genes whose expression changed due to seed development

Total 219 probe sets were identified for seed development whose expression changed at least ten-fold between good and poor quality varieties in at least one of the three condition pairs (‘Good-7 DAA’ vs ‘Poor-7 DAA’, ‘Good-14 DAA’ vs ‘Poor-14 DAA’, and ‘Good-28 DAA’ vs ‘Poor-28 DAA’) (Table 2, see Additional file 3, Figures 1 and 2B). Using blastx similarity search of the sequence of the 219 probe sets at NCBI, the putative gene function was assigned to 153 probe sets (69.9% = 153/219), hypothetical proteins to 57 (26.0% = 57/219) probe sets, and similarity of the remaining 9 probe sets was not found in the database. Out of 219, 171, 2, and 35 probe sets showed differential expression in 7, 14, and 28 DAA, respectively and the remaining 11 probe sets were differentially expressed in at least two seed development stages (Figure 2B). Further analysis revealed that out of 219, the expression of 37 probe sets was also changed due to quality (Figure 1).

Out of 153, the majority of probe sets (35.9% = 55/153) were dominated by seed storage protein genes such as gliadin (alpha-/beta-, gamma, and omega gliadins), glutenin (LMW glutenin subunit) and HMW glutenin subunit, and avenin-like. Other genes were related to grain hardness such as puroindolines and grain softness proteins, starch biosynthesis metabolism such as granule bound starch synthase-I (GBSS-I), alpha- and beta-amylase inhibitors, peroxidase, trypsin inhibitor, etc. (see Additional file 3).

Four probe sets related to genes for grain hardness and softness showed differential expression from about ten-fold (Ta.14614.1.S1_at) to about 43-fold (Ta.69.2.S1_x_at) in early stage of seed development i.e. 7 DAA and one probe set (Ta.23141.1.S1_at) showed about 22-fold differential expression at later stage of seed development i.e. 28 DAA between good and poor varieties (see Additional file 3). Grain hardness is considered as a single important trait that determines the quality of end-use products such as bread and cookies or biscuit [33]. Puroindoline genes coding puroindoline a and b are major contributor of grain hardness. If puroindoline a and b are functional, the grain is soft or both or either one of them are mutated or absent, the grain is very hard [34]. Out of five, two probe sets (Ta.69.2.S1_x_at and Ta.840.1.S1_at) represent grain softness proteins. Jolly *et al.*[35] reported the linkage of the genes of grain softness proteins to the grain hardness locus (*Ha*). However, no direct or indirect relationship of this protein to grain texture has been established [34].

The wheat microarray contains probe sets of more than 28 genes of starch metabolism. In this study, only GBSS (granule bound starch synthase) and beta-amylase showed at least 10-fold differential expression between the contrasting varieties. Beta amylase (Ta.27780.1.S1_x_at) expression was involved in quality and seed development as well as in their interactions (Additional file 3: Table S3). Its differential expression (about 60-fold) was only present in early stage of seed development (7 DAA). Beta-amylase is responsible for starch hydrolysis at the time of seed germination by releasing maltose (two glucose units) of the second α-1,4 glycosidic bonds from the non-reducing end. It accumulates during grain maturation, but remains inactive till germination.

GBSS is believed to be responsible for synthesis of amylose in seed (for review, see [36]. The content of amylose affects processing quality. GBSS mutant analysis found amylose free seed indicating that this enzyme is responsible for amylose synthesis [37]. This enzyme is found in two forms- GBSSI in endosperm and GBSSII in non-endospermic tissues [38]. In this study a high increase in differential expression of GBSS from early (about 7-fold at 7 DAA) to late maturity (about 93 fold at 28 DAA) was observed between good and poor quality varieties. The expression of the GBSS gene was involved in both qualities as well as in seed development, but not in interaction of quality and development (see Additional file 3).

Cluster analysis of the 219 probe sets showing at least ten-fold differential expression into 5 clusters identified the co-expressed genes having similar expression patterns in different seed development stages in good and poor varieties (see Additional file 5, Figure 3). Cluster analysis of the 219 probe sets grouped the probe sets in the same cluster having similar expression patterns (see Additional file 5, Figure 3). In cluster I, the probe sets showed up-regulation in good quality varieties in early stage of seed development (7 DAA) and did not show any much change at later stage of seed development. In cluster II, the majority of the probe sets showed up-regulation in good quality varieties at later stages of seed development (14 and 28 DAA). In cluster III, the probe sets showed down-regulation in good quality varieties at early stage i.e. 7 DAA (Figure 3). In clusters IV and V, the probe sets showed up-regulation in good varieties at early stage seed development i.e. 7 DAA. The key genes for processing quality, mainly seed storage protein genes were clustered in both cluster IV and V.

### Identification of candidate genes involved in interaction (quality x development)

Total 85 probe sets was identified for interaction (quality x development) showing at least ten-fold expression between good and poor varieties in at least one of the three condition pairs (‘Good-7 DAA’ vs ‘Poor-7 DAA’, ‘Good-14 DAA’ vs ‘Poor-14 DAA’, and ‘Good-28 DAA’ vs ‘Poor-28 DAA’) (Table 2, see Additional file 3, Figure 1). These probe sets were involved in development. Of them, 56 probe sets also involved in quality (Figure 1, see Additional file 3). Further analysis revealed that 68, 2, and 13 probe sets (out of the 85 probe sets) were differentially expressed in 7, 14, and 28 DAA, respectively (Figure 2C).

Cluster analysis of the 85 probe sets grouped the probe sets in the same cluster having similar expression patterns (see Additional file 5, Figure 3). In cluster I, the probe sets showed up-regulation in poor varieties at early stage of seed development (i.e. 7 DAA) and did not show any change at later stage of seed development. In cluster II, the probe sets showed down-regulation in poor varieties at early stage of seed development (i.e. 7 DAA) and did not show any change at later stage of seed development. The most of probe sets for seed storage protein genes were grouped in cluster II. In cluster III, there was a single probe set (Ta.2025.1.S1_at, oxi-reductase, a Fe (II) oxygenase family protein) showing up-regulation in poor varieties at 14 and 28 DAA (Figure 3). In cluster IV, the probe sets showed up-regulation in good varieties at later stage seed development (28 DAA). In cluster V, the majority of the probe sets showed up-regulation in good varieties at early stage of seed development.

### Spatial distribution of the expression of 110 probe sets related to processing quality across 10 development stages

**Figure 4:**
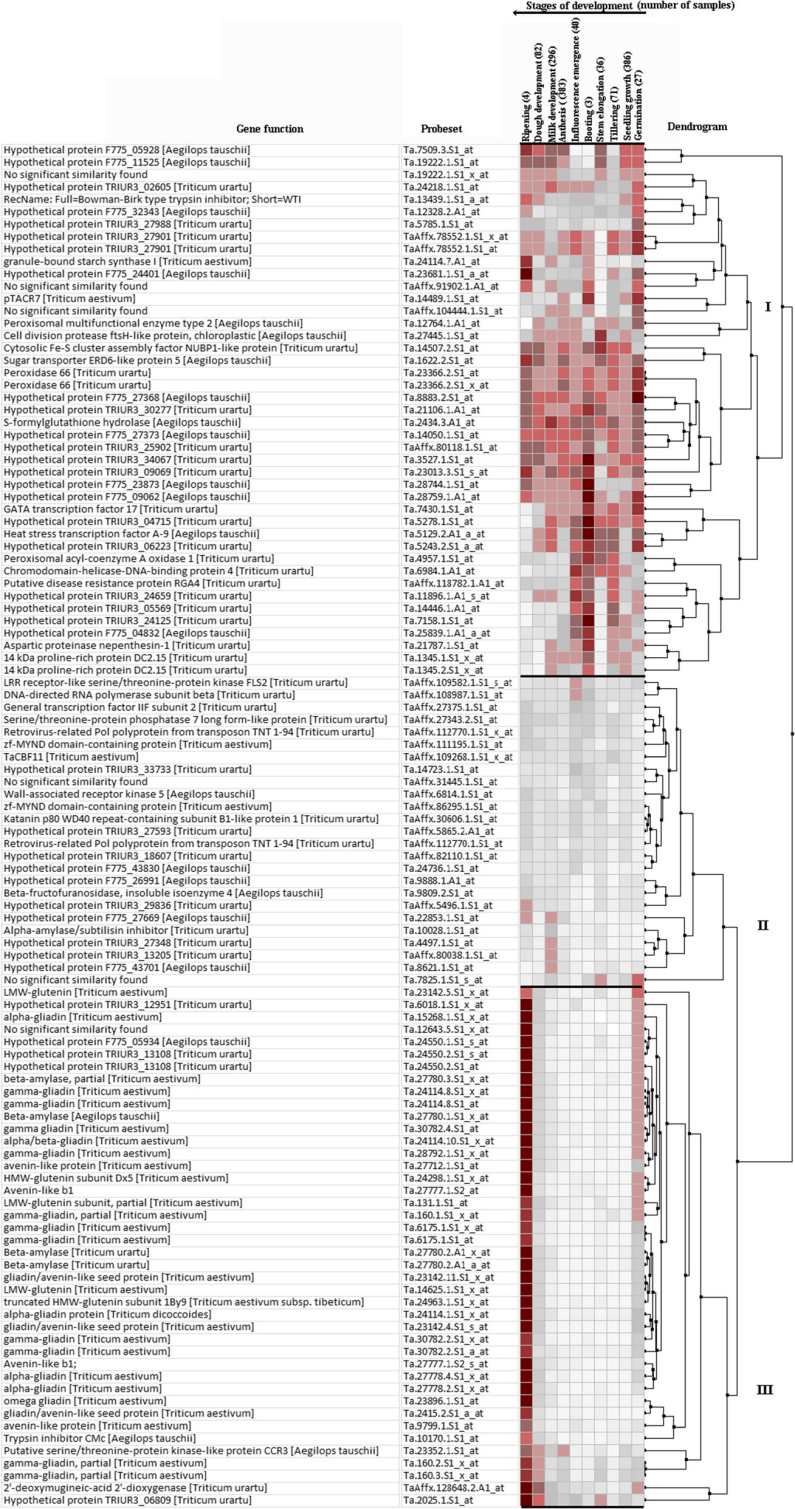
**A heat map of the 110 probe sets (identified for quality) indicating level of expression potentials in 10 development stages such as germination, seedling growth, tillering, stem elongation, booting, inflorescence emergence, anthesis, milk development, dough development, and ripening.** The expression potentials of the 110 probe sets were estimated in 1,328 samples which were available in the Affymetrix®’s *Triticum aestivum* microarray database. The darkest red color represents the highest level of probe set expression potential. The expression potential is defined as the average of the top 1% signal values across all samples for a given probe set in a given platform. The heat map was generated in Genevestigator (Nebion AG, Zurich, Switzerland).

Using gene-specific two-way ANOVA analysis, 110 probe sets were identified for processing quality showing at least 10-fold differential expression between good and poor quality varieties. The expression level of these probe sets were analysed across 1,328 samples which were available online and representing ten developmental stages including seedling, tillering, and flower development stages through meta-analysis using Genevestigator software. The heatmap produced after the analysis revealed expression potential of the individual probe sets in 10 development stages (Figure 4). Cluster analysis of the expression potential of the probe sets distinctly grouped the probe sets into three major clusters i.e. I, II and III of 43, 25, and 42 probe sets, respectively (Figure 4).

In cluster I the expression level of the 43 probe sets was global as they were present in the majority of the ten developmental stages (Figure 4). About 42% (18/43) of the probe sets was annotated with blastx and showing homology to genes related to cellular and molecular mechanisms such as granule-bound starch synthase I (GBSSI), Bowman-Birk type trypsin inhibitor, aspartic proteinase nepenthesin-1, cell division protease, peroxidase, sugar transporter, transcription factors (GATA and heat stress TF A-9), 14 KDa proline-rich protein, low temperature specific wheat gene pTACR7, peroxisomal acyl-coenzyme A oxidase 1, etc. About of 51% (22/43) of the probe sets were hypothetical proteins to *Aegilops tauschii* and *Triticum urartu* and the significant similarity was not found for the other three probe sets.

Except eight probe sets, the expression level of other 17 probe sets in cluster II was low i.e. white or grey color in the heatmap (Figure 4). Majority of the probe sets (52% = 13/25) was annotated with blastx and showed homology to kinases (LRR receptor-like serine/threonine protein kinase and wall-associated receptor kinase 5), DNA-directed RNA polymerase, serine/threonine protein phosphatase, beta-fructofuranosidase, alpha-amylase/subtilisin inhibitor, transcription factor IIF, etc. Forty percentage (10/25) of the 25 probe set was hypothetical proteins and the significant similarity was not found for the other two probe sets.

In cluster III the expression level of the probe sets were strong in ripening and the majority of them had also expression in germination stage (Figure 4). Majority of the probe sets (about 69% = 29/42) were related to seed storage protein genes such as gliadins (alpha gliadin, alpha/beta gliadin, gamma gliadin), glutenin (low molecular weight and high molecular weight glutenin subunits), and avenin like proteins. The seven probe sets were related to beta-amylase, trypsin inhibitor, serine/threonine protein kinase and deoxymugineic acid dioxygenase. Five probe sets were hypothetical proteins to *Aegilops tauschii* and *Triticum urartu*. The significant similarity was not found for the remaining one probe set.

### Spatial distribution of the expression of 110 probe sets related to processing quality across 22 tissues/organs

**Figure 5:**
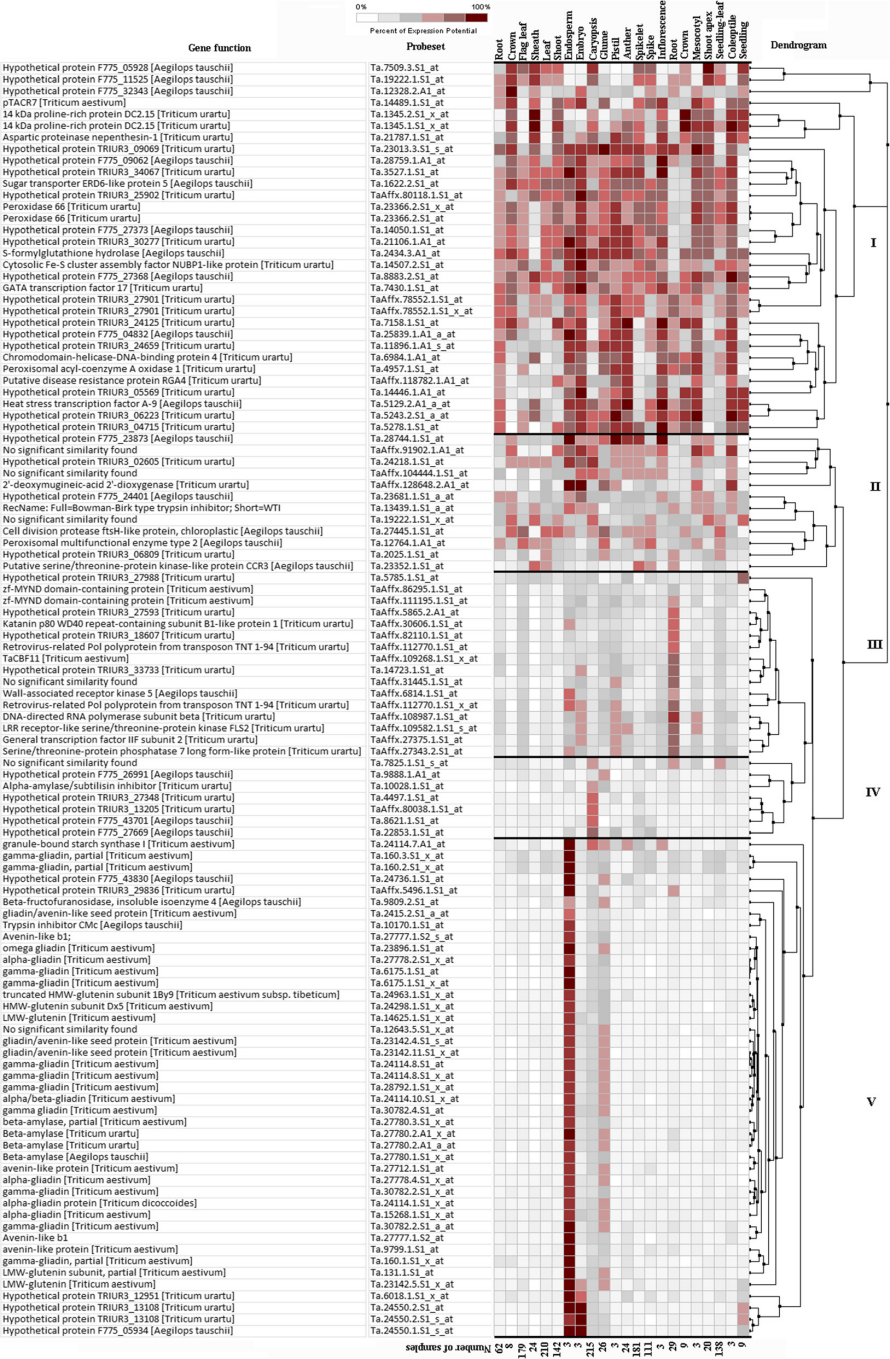
**A heat map of the 110 probe sets (identified for quality) indicating level of expression potentials in 22 wheat tissues such as endosperm, glume, caryopsis, embryo, leaf, root, coleoptile, mesocotyl, seedling, sheath, shoot, shoot apex, leaf, flag leaf, crown, inflorescence, spikelet, pistil, anther, glumes, caryopsis, endosperm, embryo.** The expression potentials of the 110 probe sets were estimated in 1,405 samples available in the Affymetrix®’s *Triticum aestivum* microarray database. The darkest red color represents the highest level of probe set expression. The expression potential is defined as the average of the top 1% signal values across all samples for a given probe set in a given platform. The heat map was generated in Genevestigator (Nebion AG, Zurich, Switzerland). ‘Root’ labelled at 16th column of the heatmap represents ‘roots of seedling’. ‘Crown’ labelled at 17^th^ column of the heatmap represents ‘Crown of seedling’.

The expression level of the 110 probe sets related to processing quality were analysed across 1,405 samples which were available online and representing 22 tissues including root, shoot, flag leaf, endosperm, embryo, coleoptile, and mesocotyl through meta-analysis using Genevestigator software. The heatmap produced after the analysis revealed expression potential of the individual probe sets in different tissues (Figure 5). Cluster analysis of the expression potential of the probe sets distinctly grouped the probe sets into three major clusters i.e. I, II, and III of 44, 23, and 43 probe sets, respectively (Figure 5), which was very similar to the clusters produced for the development stages (described in the previous section). The percentage of probe sets grouped in both clusters (Figures 4 and 5) was very similar i.e. 93.2% (cluster I), 95.7% (cluster II), and 90.7% (cluster III). Briefly, the expression level of the probe sets in cluster I was very strong (red color in the heatmap in Figure 5) and global as they were present in the most of the 22 tissues. Except a few probe sets, the expression level of the majority of the probe sets in cluster II and III were specific to one to two tissues (Figure 5). Majority of the probe sets in cluster III were related to seed storage protein genes such as gliadins (alpha gliadin, alpha/beta gliadin, gamma gliadin), glutenin (low molecular weight and high molecular weight glutenin subunits), and avenin like proteins.

The heatmaps providing information on spatial distribution of the genes would assist in designing functional genomics experiments specific to a particular growth stage or tissue. The correct interpretation of results is very important for formulating new hypothesis and models, and designing proper experiments to test them [39].

### Pathway analysis of the 35,472 probe sets

The 35,472 probe sets (see Additional file 1) which passed the gene-specific two-way ANOVA (corrected *p* value of 0.05) were mapped to metabolism pathways by using MapMan software on the *Triticum aestivum* mapping data, “Taes_Affy_0709”. The genes of several metabolic pathways such as starch (AGPase, starch synthase, starch branching enzymes, starch debranching enzymes, and starch transporter), myo-inositol, sugar and sugar-alcohol, and cell wall were up-regulated at early stage of seed development (7 DAA) in good quality varieties (Figure A in Additional file 6). The genes of photo systems (light reactions) and Calvin cycle were down-regulated in early stage of seed development (7 DAA) in good quality varieties (Figure A in Additional file 6). The activities of other metabolic pathway genes were more differentiated at 7 DAA between good and poor varieties (Figure A in Additional file 6), but less at later stages (14 and 28 DAA) of seed development (Figures B and C in Additional file 6). This indicates that genes for seed development and processing quality are regulated at early stage of seed development.

### Validation of differential expression by quantitative RT-PCR

**Figure 6:**
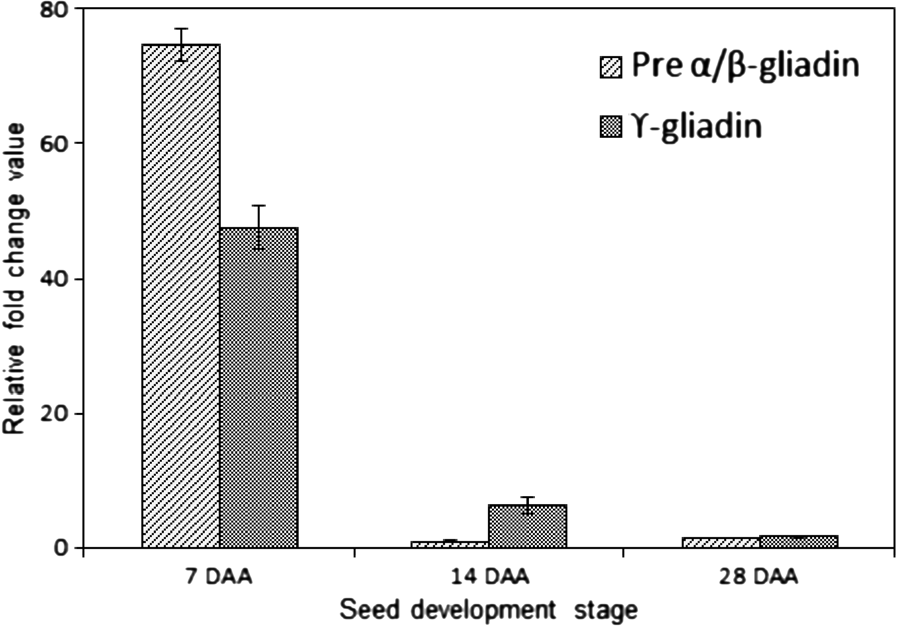
**Validation of differential expression (fold change) of two randomly chosen candidate genes (pre-α/β-gliadin and γ-Gliadin) at three seed development stages using qRT-PCR.** The validation was done between a good (C306) and a poor (Sonalika) chapatti quality varieties in 7, 14, and 28 days after anthesis (DAA) of seed developmental stage through quantitative real time PCR (qRT-PCR). Y-axis represents fold change in differential expression of genes in the wheat variety, C306 in comparison to the wheat variety, Sonalika. X-axis represents three seed development stages i.e. 7, 14, and 28 DAA. The expression data were normalized to that of a control gene, ADP ribosylation factor, ARF. qRT-PCR data analysis was done following Livak and Schmitteng (2001) [46].

The expression profiles of a few randomly chosen genes were validated by quantitative RT-PCR. A set of reference genes has been recently proposed for common wheat such as gene for cell division control protein, AAA-superfamily of ATPases (CDC, Ta.54227), ADP-ribosylation factor, ARF (ADPRF, Ta.2291), and RNase L inhibitor-like protein (RLI, Ta.2776) [40]. For this study one house-keeping gene such as ARF was used as a reference gene for quantitative validation of the expression data. The two differentially expressed genes, pre-α/β-gliadin and γ-gliadin, were randomly chosen for validation of the microarray data (Tables 5 and 6, Figure 6). The individual C_T_ values of the house-keeping gene at the three seed development stages, 7, 14, and 28 DAA, in the two diverse chapatti making varieties, C306 and Sonalika were used for normalization. The two target genes showed similar difference in their expressions through seed developmental stages between the varieties, which are largely in agreement with the microarray data (Table 6). For example, the expressions of pre-α/β-gliadin and γ-gliadin genes were very high in the variety C306 than the variety Sonalika at early stage seed development, 7 DAA (Figure 6, Table 6). Hence, the microarray data were validated by qualitative PCR.

**Table 5:** Nucleotide sequences of forward and reverse primers of two randomly chosen candidate genes for processing quality (pre-α/β-gliadin and γ-Gliadin) and one control (ADP ribosylation factor, ARF) used for their validations of differential expression between good and poor quality varieties using quantitative real time PCR (qRT-PCR)

**Gene**	**Forward primer (5′-3′)**	**Tm (°C)**	**Reverse primer (5′-3′)**	**Tm (°C)**	**Product size (bp)**
ADP ribosylation factor (ARF)	TGATAGGGAACGTGTTGTTGAGGC	57.4	AGCCAGTCAAGACCCTCGTACAAC	59.1	200
Pre-α/β-gliadin	GACCTTTCTCATCCTTGTCCTCCT	57.4	CTGTGAATATGGTAGTTGCGGCTG	57.4	265
γ-Gliadin	TCTCTACAACAACAGATGAACCCCTG	58.0	GCCTTGTTGTTGTTCTTGCTGCATG	57.7	225

**Table 6:** Fold change values of the validated (by qRT-PCR) pre-α/β-gliadin and γ-gliadin genes between good and poor quality varieties at three seed development stages using qRT-PCR and microarrays

Gene/seed development stage*	**Fold change values in gene expression between C306 and Sonalika**	
	**qRT-PCRs**	**Affymetrix wheat microarrays**
**pre α/β-gliadin**		
7 DAA	74.7	100.7
14 DAA	1.0	-1.0
28 DAA	1.4	-1.2
**γ-gliadin**		
7 DAA	47.6	38.8
14 DAA	6.4	-1.4
28 DAA	1.7	-2.0

*Seed development stages at 7 DAA (days after anthesis), 14 DAA, and 28 DAA.

## Conclusion

Genome-wide transcriptome analysis with help of two-way ANOVA in developing caryopsis identified a substantial number of differentially (at least 10-fold) expressed genes in two diverse sets of Indian wheat varieties for chapatti quality. Many key processing quality related genes such as different subunits of glutenin and gliadins, puroindolines, grain softness protein, alpha and beta amylases, proteases, peroxidase, GBSS, etc. were identified. In addition, many other candidate genes related to cellular and molecular functions were also identified. The ANOVA analysis revealed that the expression of majority of the candidate genes for good chapatti was involved in interaction of quality and development. Most of these probe sets showed differential expression at early stage of seed development i.e. temporal expression. Meta-analysis revealed that the majority of the genes expressed in one or a few growth stages indicating spatial distribution of their expressions. The differential expression of the two candidate genes, pre-α/β-gliadin and γ-gliadin between good and poor chapatti quality varieties was validated by quantitative real time PCR. Therefore, this study able to identify several known processing quality related genes and many additional candidate genes for good chapatti and their interactions with development and temporal and spatial distributions. Gene identified for processing quality and information on temporal and spatial distributions of their expressions would be useful for designing programs for improvement of processing quality either by changing expression (over expression or down regulation) of genes or development of single nucleotide polymorphisms (SNPs) markers using bi-parental mapping populations for molecular breeding.

## Methods

### Plant materials

Two traditionally known good chapatti making varieties, C306 and Lok1 and two poor chapatti making varieties, Sonalika and WH291 were used for transcriptome studies. The detailed information on the varieties described in Additional file 7. These varieties were grown in three replicates at the Research Farm of National Agri-Food Biotechnology Institute (NABI) Mohali, India during 2010-11. The main individual spikes of the three biological replications of each of the four varieties were tagged at first day of anthesis. The tagged spikes were harvested at three main seed developmental stages i.e. 7, 14, and 28 DAA, immediately frozen in liquid nitrogen, and stored at -80°C for RNA extraction.

### RNA extraction

Total RNA was extracted from about 10 caryopsis of central parts of the harvested spikes using the TRIzol reagent (Life Technologies, NY, USA) and purified onto RNeasy Mini columns (Qiagen, Hilden, Germany). The extracted RNA was quantified on a NanoQuant, Infinite M200 PRO (Tecan, Mannedorf, Switzerland) and the quality was checked onto 1.5% agarose gels. RNA integrity number (RIN) of the extracted RNA was estimated on Agilent’s 2100 Bioanalyzer using the Agilent RNA 6000 Nano Kit (Agilent Technologies, Santa Clara, USA). The images of RIN values of the extracted RNAs were present in Additional file 8. The good quality RNA samples (RIN value >7.0) were stored at -80°C for microarray experiments.

### Wheat microarrays

The GeneChip® Wheat Genome Array (Affymetrix, Santa Clara, USA), a 3’ *in vitro* transcription (3′ IVT) expression array containing 61,290 probe sets representing about 25 K unigenes was used for gene expression analysis. The sequence and annotation information of the probe sets on the array are available at http://www.affymetrix.com.

### Microarray hybridization and scanning

RNA labelling and microarray hybridization on wheat genome arrays were performed according to its manual. The arrays were processed on an Affymetrix GeneChip® Fluidics Station 450 by running fluidic script EukGE-WS2v5-450. The hybridized arrays were scanned on a Affymetrix GeneChip® Scanner 3000. Hybridization quality of the microarrays was verified by scaling factor, overall hybridization rate, and signal strength of several bacterial spike controls.

### Microarray data analysis

i) Normalization

The expression console of Affymetrix’s GeneChip Command Console (AGCC) software was used for preliminary data analysis of the scanned arrays. The image files were created as *.dat file. The software computed cell intensity data of probe sets and their positional values (*.cel file) from the image file (*.dat). The normalization of the signal intensities of the probe sets (taking two types of files, *.cel and *.ARR) was carried out using the Robust Multi-array normalization algorithm (RMA) implemented in GeneSpring’s Multi-omic analysis (version 11.5.1, build-138755) software (Agilent Technologies, Santa Clara, USA). The RMA normalized values of the probe sets were used for statistical analysis.

ii) Two-way ANOVA analysis

Two levels of categories (quality and developmental stage) were made for two-way ANOVA analysis of the gene expression data. For quality category, the four samples were divided into two groups- good varieties (i.e. good chapatti) and poor varieties (poor chapatti). For seed developmental stage category, each variety was divided into three groups - 7, 14, and 28 DAA. Therefore, six pairwise conditions (‘Good-7 DAA’, ‘Poor-7 DAA’, ‘Good-14 DAA’, ‘Poor-14 DAA’, ‘Good-28 DAA’, and ‘Poor-28 DAA’) were made for analysis. Variation in the expression data was partitioned into three groups, namely quality (genotype), developmental stage, and interaction of quality and developmental stage. ‘Asymptotic’ *p* value computation and the Benjamini-Hochberg false discovery rate (FDR) for multiple test correction (corrected *p* < 0.05) were used for the analysis. The two-ANOVA analysis was conducted in GeneSpring software.

iii) Fold change analysis

The probe sets satisfying the corrected *p* value in two-way ANOVA analysis were used for fold change analysis in GeneSpring software. The probe sets showing at least two-fold and ten-fold differential expressions between good and poor groups were used for further analysis.

iv) Cluster analysis of gene expression data

The probe sets in each of the three groups (quality, developmental stage, and interaction) as mentioned above were grouped by assuming five clusters by estimating pairwise Euclidean distance between genes, 50 iterations, and K-means algorithm implemented in GeneSpring software. The probe sets were clustered for identification of the probe sets with similar expression profiles.

v) Meta-analysis of the differentially expressed genes

Affymetrix® wheat microarray expression data conducted in a wide variety of growth stages and tissues were available online. These data were good resources for meta-analysis to study spatial distribution of the gene expression data in different growth stages and tissues/organs. This information would assist in designing functional genomics experiments specific to a particular growth stage or tissue. The spatial distribution of the differentially expressed genes (at least 10-fold between good and poor varieties) was studied in ten growth stages, namely, germination, seedling growth, tillering, stem elongation, booting, inflorescence emergence, anthesis, milk development, dough development, and ripening. Their spatial distributions were also studied in 22 tissues/organs; namely, shoot apex, crown, sheath, caryopsis, seedling, spike, spikelet, flag leaf, shoot, shoot-leaf, seedling-leaf, crown, roots, seedling-root, glume, coleoptile, anther, mesocotyl, embryo, endosperm, inflorescence, and pistil. Their spatial distributions were represented in form of heatmaps. Meta-analysis was conducted using Genevestigator software which retrieved the expression data from the web [41,42]. Further, the expression data was clustered through hierarchical clustering on the estimated Euclidean distance matrix using optimal leaf-ordering implemented in the software.

vi) Functional analyses of the differentially expressed data

The differential expression data of the 35,472 probe sets satisfying the corrected *p* value of 0.05 in two-way ANOVA (See Additional file 1) was assigned to metabolic pathways by MapMan software (version 3.5.1.R2) using the *Triticum aestivum* mapping data “Taes_Affy_0709” [43-45].

### Validation of differential expression by quantitative RT-PCR

Quantitative real time PCR (qRT-PCR) was performed for validation of a few differentially expressed genes. Five microgram of the extracted RNA was treated with TURBO DNase™ (Ambion, Life Technologies, NY, USA) before cDNA synthesis according to the manufacturer manual. The SuperScript*®* III First-Strand Synthesis kit (Invitrogen, Life Technologies, NY, USA) was used for cDNA synthesis using oligodT (18-mer). The reaction mixture was incubated at 50°C for 50 min, then at 85°C for 5 min, and chilled immediately on ice. The mixture was diluted with nuclease free water to the final concentration of 20 ng/μl cDNAs. The primers used for PCR were designed to the same region harbouring the microarray probes using Primer Express software (Applied Biosystems, Forster City, CA, USA) (Tables 7 and 5). qRT-PCR was done in triplicates using SYBR Green fluorescence dye in a qRT-PCR machine (7500 Fast Real-Time PCR System, Applied Biosystems). Each reaction was prepared using 5 μl of 2X QuantiTect SYBR Green (Qiagen, USA), 1 μl of 20 ng/μl cDNA, and 1 pmol/μl each of forward and reverse primers, in a total volume of 10 μl. The cycling conditions were: 30 sec at 95°C, followed by 45 cycles of 95°C for 30 sec and 60°C for 1 min. To evaluate the presence of non-specific PCR products and primer dimers, the amplified PCR products were run at temperature ramp of 95°C for 15 sec, 60°C for 15 sec, followed by 20 min of slow ramp from 60°C to 95°C. The threshold cycles (Ct) of each target genes were normalized in each wheat varieties with Ct of the internal control (wheat ARF gene: AB050957.1). Normalization and quantification of relative changes in gene expression or fold changes between one good and one poor varieties were calculated by using the following formula: FC = 2^-Δ (ΔCt)^[46].

**Table 7:** Detail of two randomly chosen candidate genes for processing quality (pre-α/β-gliadin and γ-Gliadin) and one control (ADP ribosylation factor, ARF) used for validation of their differential expressions between good and poor quality varieties using quantitative real time PCR (qRT-PCR)

**Gene**	**NCBI ID**	**Probe ID**	**Unigene ID**	**Transcription ID (http://aranet.mpimp-golm.mpg.de/wheatnet)**
ADP ribosylation factor (ARF)	AB050957.1	Ta.2291.1.S1_x_at	Ta.2291	UniRef90_Q677H6
Pre-α/β-gliadin	K03076.1	Ta.27778.4.S1_x_at	Ta.24085	UniRef90_B8XU31
γ-Gliadin	FJ006618.1	Ta.24114.14.S1_x_at	Ta.24114.1	UniRef90_P21292

All microarray experimental data have been deposited in the NCBI’s Gene Expression Omnibus (GEO) database, http://www.ncbi.nlm.nih.gov/geo/, with accession number **GSE53606**.

## Competing interests

The authors’ declare that they have no competing interests.

## Authors’ contribution

AS conducted experimental works and data analysis. SM helped in computational analysis. MS helped in a part of experimental works. AC and RT helped in critical review of the manuscript. JR helped in experiment designing, data analysis, and manuscript writing. All authors contributed to writing the manuscript. All authors read and approved the final manuscript.

## Supplementary Material

Additional file 1 Details of 35,472 probe sets identified through gene specific two-way ANOVA and their fold change values. Click here for file

Additional file 2 **Details of the probe sets identified for quality, seed development, and interaction (quality and seed development) through gene specific two-way ANOVA.** Sheet 1: Detail of 3,126 probe sets whose expression changed due to quality. Sheet 2: Detail of 34,604 probe sets whose expression changed due to seed development. Sheet 3: Detail of 1,732 probe sets whose expression changed due to interaction (quality x development). Click here for file

Additional file 3 **Gene-specific two-way ANOVA identified a total of 236 probe sets for quality, seed development, and interaction (quality x seed development) showing at least 10-fold differential expression between good and poor quality varieties in three seed developmental stages (i.e. 7, 14, and 28 days after anthesis, DAA).** Among a total of 236 probe sets, 110, 219, and 85 probe sets were involved for quality, seed development, and interaction (quality x seed development), respectively. Gene function was assigned to the probe sets using blastx at NCBI web site. (+ = involved, - = not involved, up = up-regulated, down = down regulated). Click here for file

Additional file 4 **Pairwise comparison of differential expression in three seed developmental stages [7, 14, and 28 days after anthesis (DAA)] of the probe sets of seed storage protein genes which were identified for quality in the four Indian wheat varieties.** C306 and Lok1 were good and Sonalika and WH291 were poor chapatti making varieties. Click here for file

Additional file 5 **Details of the probe sets in each of the five clusters identified through cluster analysis for quality, seed development, and interaction (quality x seed development).** Sheet 1: Details of five clusters of 110 probe sets whose expressions changed due to quality between good and poor quality wheat varieties. Sheet 2: Details of five clusters of 219 probe sets whose expressions changed due to seed development namely 7, 14 and 28 days after anthesis (DAA). Sheet 3: Details of five clusters of 85 probe sets whose expressions changed due to interaction (quality x seed development). Click here for file

Additional file 6 **Display of genes of 35,472 probe sets to metabolic pathways using MapMan software (version 3.5.1R2)**[43-45]. The transcript levels of genes in several metabolic pathways were visualized by colour scales (+4.5 red to blue –4.5), where red and blue represents an increase and decrease of expression (log_2_ of fold change value), respectively in good quality varieties in comparison to poor making varieties in 7 days after anthesis (Top figure **A**), 14 DAA (Middle figure **B**), and 28 DAA (Last figure **C**). Click here for file

Additional file 7 Detail of the four India wheat varieties used in transcriptome studies. Click here for file

Additional file 8 Quality evaluations of the extracted RNAs from three replications of each of the four Indian wheat varieties (C306, Lok1, Sonalika, and WH291) at three seed development stages [7, 14, and 28 days after anthesis (DAA)]. Click here for file
