# Supplementary material for: Genome-wide transcriptome study in wheat identified candidate genes related to processing quality, majority of them showing interaction (quality x development) and having temporal and spatial distributions
Source: BMC Genomics. 2014 Jan 16;15:29. doi: 10.1186/1471-2164-15-29 (PMC3897974; doi:10.1186/1471-2164-15-29)
Supplement: Additional file 3 — Gene-specific two-way ANOVA identified a total of 236 probe sets for quality, seed development, and interaction (quality x seed development) showing at least 10-fold differential expression between good and poor quality varieties in three seed developmental stages (i.e. 7, 14, and 28 days after anthesis, DAA). Among a total of 236 probe sets, 110, 219, and 85 probe sets were involved for quality, seed development, and interaction (quality x seed development), respectively. Gene function was assigned to the probe sets using blastx at NCBI web site. (+ = involved, - = not involved, up = up-regulated, down = down regulated). [file 1471-2164-15-29-S3.docx]

**Additional file 3:** Gene-specific two-way ANOVA identified a total of 236 probe sets showing at least 10-fold differential expression between good and poor wheat varieties at three seed development stages (i.e. 7, 14, and 28 days after anthesis, DAA). Among a total of 236 probe sets, 110 probe sets were involved in quality, 219 probe sets in seed developmental stage, and 85 probe sets in interaction of quality and seed developmental stage. The probe sets showed at least 10-fold differential gene expression in at least one of the three seed developmental stages. Gene function was assigned to the probe sets using blastx at NCBI web site. (+ = involved, - = not involved, up = up-regulation, down = down regulation)

| **S. No.** | **Probeset ID** | **Unigene** | **Potential gene function** | **Quality** | **Seed developmental stage** | **Quality x seed developmental stage** | **7 DAA** | | **14 DAA** | | **28 DAA** | |
| --- | --- | --- | --- | --- | --- | --- | --- | --- | --- | --- | --- | --- |
|  |  |  |  |  |  |  | **Fold change** | **Regulation** | **Fold change** | **Regulation** | **Fold change** | **Regulation** |
| 1 | Ta.24114.10.S1_x_at | Ta.28482 | alpha/beta-gliadin [Triticum aestivum] | + | + | + | 54.1 | up | 1.1 | up | 1.1 | up |
| 2 | Ta.24114.1.S1_x_at | Ta.67647 | alpha-gliadin protein [Triticum dicoccoides] | + | + | + | 171.4 | up | 1 | up | 1.2 | down |
| 3 | Ta.131.1.S1_at | Ta.131 | LMW-glutenin subunit, partial [Triticum aestivum] | + | + | + | 58.9 | up | 1.1 | up | 1.2 | up |
| 4 | Ta.23142.5.S1_x_at | Ta.62234 | LMW-glutenin [Triticum aestivum] | + | + | + | 57.8 | up | 1 | up | 1.3 | up |
| 5 | Ta.14625.1.S1_x_at | Ta.55438 | LMW-glutenin [Triticum aestivum] | + | + | + | 106 | up | 1.1 | down | 1.2 | down |
| 6 | Ta.24963.1.S1_x_at | Ta.24963 | truncated HMW-glutenin subunit 1By9 [Triticum aestivum subsp. tibeticum] | + | + | + | 60.4 | up | 1.1 | down | 1.1 | up |
| 7 | Ta.23142.11.S1_x_at | - | gliadin/avenin-like seed protein [Triticum aestivum] | + | + | + | 124.1 | up | 1.1 | up | 1.1 | down |
| 8 | Ta.23142.4.S1_s_at | - | gliadin/avenin-like seed protein [Triticum aestivum] | + | + | + | 175.9 | up | 1 | up | 1 | up |
| 9 | Ta.28792.1.S1_x_at | Ta.66066 | gamma-gliadin [Triticum aestivum] | + | + | + | 146.9 | up | 1 | up | 2.2 | up |
| 10 | Ta.24114.8.S1_x_at | Ta.55042 | gamma-gliadin [Triticum aestivum] | + | + | + | 194.8 | up | 1.1 | up | 1.8 | up |
| 11 | Ta.30782.4.S1_at | Ta.54474 | gamma gliadin [Triticum aestivum] | + | + | + | 98 | up | 1 | up | 1 | up |
| 12 | Ta.27780.1.S1_x_at | Ta.54284 | Beta-amylase [Aegilops tauschii] | + | + | + | 58.6 | up | 1.2 | down | 1.1 | down |
| 13 | Ta.27777.1.S2_s_at | Ta.63018 | Avenin-like b1; | + | + | + | 122.6 | up | 1.1 | up | 1 | up |
| 14 | Ta.27712.1.S1_at | Ta.54540 | avenin-like protein [Triticum aestivum] | + | + | + | 86.6 | up | 1.1 | up | 1.3 | up |
| 15 | Ta.27778.4.S1_x_at | Ta.24085 | alpha-gliadin [Triticum aestivum] | + | + | + | 284.7 | up | 1.3 | up | 1.2 | down |
| 16 | Ta.15268.1.S1_x_at | Ta.63498 | alpha-gliadin [Triticum aestivum] | + | + | + | 110.3 | up | 1.1 | up | 1.1 | up |
| 17 | Ta.6175.1.S1_at | Ta.35463 | gamma-gliadin [Triticum aestivum] | + | + | + | 105.9 | up | 1.2 | up | 1.5 | down |
| 18 | Ta.12643.5.S1_x_at | Ta.55449 | No significant similarity found | + | + | + | 92.5 | up | 1.2 | up | 1 | up |
| 19 | Ta.6175.1.S1_x_at | Ta.35463 | gamma-gliadin [Triticum aestivum] | + | + | + | 80.9 | up | 1.4 | up | 1.2 | down |
| 20 | Ta.27780.3.S1_x_at | Ta.54269 | beta-amylase, partial [Triticum aestivum] | + | + | + | 72 | up | 1 | down | 1.1 | up |
| 21 | Ta.27777.1.S2_at | --- | Avenin-like b1 | + | + | + | 57.6 | up | 1 | up | 1.3 | up |
| 22 | Ta.24298.1.S1_x_at | Ta.54997 | HMW-glutenin subunit Dx5 [Triticum aestivum] | + | + | + | 56.7 | up | 1.1 | down | 1 | up |
| 23 | Ta.30782.2.S1_x_at | Ta.54480 | gamma-gliadin [Triticum aestivum] | + | + | + | 51.3 | up | 1.2 | up | 1.3 | up |
| 24 | Ta.23013.3.S1_s_at | Ta.33534 | Hypothetical protein TRIUR3_09069 [Triticum urartu] | + | + | + | 31.5 | down | 1.4 | down | 1.4 | down |
| 25 | Ta.24550.2.S1_s_at | Ta.54459 | Hypothetical protein TRIUR3_13108 [Triticum urartu] | + | + | + | 28.6 | up | 1.4 | down | 1.2 | down |
| 26 | Ta.24550.2.S1_at | --- | Hypothetical protein TRIUR3_13108 [Triticum urartu] | + | + | + | 21.1 | up | 1.5 | down | 1.1 | down |
| 27 | Ta.24550.1.S1_s_at | Ta.54459 | Hypothetical protein F775_05934 [Aegilops tauschii] | + | + | + | 16.7 | up | 1.4 | down | 1.2 | down |
| 28 | TaAffx.78552.1.S1_at | Ta.55330 | Hypothetical protein TRIUR3_27901 [Triticum urartu] | + | + | + | 14.7 | up | 1.9 | up | 3.4 | up |
| 29 | Ta.9888.1.A1_at | Ta.9888 | Hypothetical protein F775_26991 [Aegilops tauschii] | + | + | + | 12.7 | up | 1.1 | up | 1.1 | up |
| 30 | Ta.21787.1.S1_at | Ta.21787 | Aspartic proteinase nepenthesin-1 [Triticum urartu] | + | + | + | 12.5 | down | 1 | down | 1.1 | up |
| 31 | Ta.1345.2.S1_x_at | Ta.55121 | 14 kDa proline-rich protein DC2.15 [Triticum urartu] | + | + | + | 12.1 | down | 1.1 | down | 1 | up |
| 32 | TaAffx.80038.1.S1_at | --- | Hypothetical protein TRIUR3_13205 [Triticum urartu] | + | + | + | 11.9 | down | 1.4 | up | 1.1 | up |
| 33 | Ta.1345.1.S1_x_at | Ta.55121 | 14 kDa proline-rich protein DC2.15 [Triticum urartu] | + | + | + | 11.1 | down | 1 | up | 1.1 | down |
| 34 | Ta.8621.1.S1_at | Ta.8621 | Hypothetical protein F775_43701 [Aegilops tauschii] | + | + | + | 10.8 | down | 1.2 | up | 1.1 | up |
| 35 | Ta.6018.1.S1_x_at | Ta.54198 | Hypothetical protein TRIUR3_12951 [Triticum urartu] | + | + | + | 10.7 | up | 1.1 | up | 1 | up |
| 36 | Ta.4497.1.S1_at | --- | Hypothetical protein TRIUR3_27348 [Triticum urartu] | + | + | + | 10.3 | down | 1.2 | up | 1.1 | up |
| 37 | Ta.22853.1.S1_at | --- | Hypothetical protein F775_27669 [Aegilops tauschii] | + | + | + | 10.1 | down | 2.8 | down | 1.1 | up |
| 38 | Ta.27778.2.S1_x_at | - | alpha-gliadin [Triticum aestivum] | + | + | + | 209.5 | up | 1.5 | up | 1.8 | down |
| 39 | Ta.24114.8.S1_at | Ta.65881 | gamma-gliadin [Triticum aestivum] | + | + | + | 168.9 | up | 1 | up | 1.9 | up |
| 40 | Ta.27780.2.A1_a_at | Ta.54269 | Beta-amylase [Triticum urartu] | + | + | + | 156.1 | up | 1 | down | 1.2 | down |
| 41 | Ta.27780.2.A1_x_at | Ta.54269 | Beta-amylase [Triticum urartu] | + | + | + | 122.4 | up | 1 | up | 1.2 | down |
| 42 | Ta.2025.1.S1_at | - | Hypothetical protein TRIUR3_06809 [Triticum urartu] | + | + | + | 1.6 | down | 86.3 | down | 1727.5 | down |
| 43 | Ta.7825.1.S1_s_at | Ta.30558 | No significant similarity found | + | + | + | 1 | up | 13.5 | up | 1.2 | up |
| 44 | Ta.5785.1.S1_at | Ta.5785 | Hypothetical protein TRIUR3_27988 [Triticum urartu] | + | + | + | 1.1 | up | 1.1 | up | 14.3 | up |
| 45 | TaAffx.111195.1.S1_at | --- | zf-MYND domain-containing protein [Triticum aestivum] | + | + | + | 1.7 | down | 3.5 | up | 13.3 | up |
| 46 | TaAffx.112770.1.S1_x_at | --- | Retrovirus-related Pol polyprotein from transposon TNT 1-94 [Triticum urartu] | + | + | + | 2.6 | down | 6.4 | up | 12.3 | up |
| 47 | TaAffx.27343.2.S1_at | --- | Serine/threonine-protein phosphatase 7 long form-like protein [Triticum urartu] | + | + | + | 1.3 | down | 2.1 | up | 11.5 | up |
| 48 | TaAffx.27375.1.S1_at | --- | General transcription factor IIF subunit 2 [Triticum urartu] | + | + | + | 2.4 | down | 5.1 | up | 11.4 | up |
| 49 | Ta.14723.1.S1_at | Ta.14723 | Hypothetical protein TRIUR3_33733 [Triticum urartu] | + | + | + | 1.7 | down | 3.5 | up | 11.3 | up |
| 50 | TaAffx.6814.1.S1_at | --- | Wall-associated receptor kinase 5 [Aegilops tauschii] | + | + | + | 1.2 | down | 1.9 | up | 11 | up |
| 51 | TaAffx.112770.1.S1_at | --- | Retrovirus-related Pol polyprotein from transposon TNT 1-94 [Triticum urartu] | + | + | + | 1.7 | down | 5.4 | up | 10.7 | up |
| 52 | TaAffx.118782.1.A1_at | Ta.57980 | Putative disease resistance protein RGA4 [Triticum urartu] | + | + | + | 2.8 | down | 1.7 | down | 10.7 | down |
| 53 | TaAffx.109582.1.S1_s_at | Ta.36406 | LRR receptor-like serine/threonine-protein kinase FLS2 [Triticum urartu] | + | + | + | 3.7 | down | 9.2 | up | 10.3 | up |
| 54 | TaAffx.109268.1.S1_x_at | --- | TaCBF11 [Triticum aestivum] | + | + | + | 1.2 | down | 2.1 | up | 10 | up |
| 55 | TaAffx.108987.1.S1_at | --- | DNA-directed RNA polymerase subunit beta [Triticum urartu] | + | + | + | 2 | down | 5.7 | up | 15.2 | up |
| 56 | Ta.12764.1.A1_at | --- | Peroxisomal multifunctional enzyme type 2 [Aegilops tauschii] | + | + | + | 1.5 | down | 1.3 | down | 14.5 | down |
| 57 | Ta.160.3.S1_x_at | Ta.62230 | gamma-gliadin, partial [Triticum aestivum] | + | + | - | 13.6 | up | 3.6 | up | 3.5 | up |
| 58 | Ta.160.2.S1_x_at | Ta.62230 | gamma-gliadin, partial [Triticum aestivum] | + | + | - | 13.3 | up | 3.8 | up | 3.5 | up |
| 59 | Ta.160.1.S1_x_at | Ta.160 | gamma-gliadin, partial [Triticum aestivum] | + | + | - | 14.6 | up | 1.8 | up | 2.7 | up |
| 60 | Ta.24114.7.A1_at | Ta.30989 | granule-bound starch synthase I [Triticum aestivum] | + | + | - | 6.7 | down | 26.9 | down | 92.8 | down |
| 61 | Ta.24736.1.S1_at | --- | Hypothetical protein F775_43830 [Aegilops tauschii] | + | + | - | 550.2 | up | 31.2 | up | 8 | up |
| 62 | Ta.7158.1.S1_at | Ta.7158 | Hypothetical protein TRIUR3_24125 [Triticum urartu] | + | + | - | 108.1 | up | 6.8 | up | 4.5 | up |
| 63 | Ta.13439.1.S1_a_at | Ta.13439 | RecName: Full=Bowman-Birk type trypsin inhibitor; Short=WTI | + | + | - | 84.5 | up | 2.1 | up | 3 | up |
| 64 | Ta.6984.1.A1_at | Ta.66222 | Chromodomain-helicase-DNA-binding protein 4 [Triticum urartu] | + | + | - | 58 | down | 7 | down | 16.9 | down |
| 65 | TaAffx.104444.1.S1_at | --- | No significant similarity found | + | + | - | 47.5 | down | 5.4 | down | 4.4 | down |
| 66 | Ta.9799.1.S1_at | Ta.9799 | avenin-like protein [Triticum aestivum] | + | + | - | 45 | up | 2.7 | up | 2.9 | up |
| 67 | Ta.23896.1.S1_at | --- | omega gliadin [Triticum aestivum] | + | + | - | 33.1 | up | 1.4 | up | 1.5 | up |
| 68 | Ta.10170.1.S1_at | --- | Trypsin inhibitor CMc [Aegilops tauschii] | + | + | - | 32.9 | up | 1.2 | up | 1.7 | up |
| 69 | Ta.10028.1.S1_at | --- | Alpha-amylase/subtilisin inhibitor [Triticum urartu] | + | + | - | 30.9 | up | 5.3 | up | 1.1 | up |
| 70 | Ta.19222.1.S1_at | Ta.13926 | Hypothetical protein F775_11525 [Aegilops tauschii] | + | + | - | 29.8 | up | 6.9 | up | 5.3 | up |
| 71 | Ta.30782.2.S1_a_at | Ta.50489 | gamma-gliadin [Triticum aestivum] | + | + | - | 28.2 | up | 1.2 | up | 1.5 | up |
| 72 | Ta.25839.1.A1_a_at | --- | Hypothetical protein F775_04832 [Aegilops tauschii] | + | + | - | 26.6 | up | 4.6 | up | 24.9 | up |
| 73 | Ta.4957.1.S1_at | Ta.4957 | Peroxisomal acyl-coenzyme A oxidase 1 [Triticum urartu] | + | + | - | 26.6 | down | 3.6 | down | 7.9 | down |
| 74 | TaAffx.80118.1.S1_at | --- | Hypothetical protein TRIUR3_25902 [Triticum urartu] | + | + | - | 20.1 | down | 5.8 | down | 32.6 | down |
| 75 | TaAffx.5496.1.S1_at | --- | Hypothetical protein TRIUR3_29836 [Triticum urartu] | + | + | - | 16.7 | up | 10.6 | up | 2.9 | up |
| 76 | TaAffx.128648.2.A1_at | Ta.35424 | 2'-deoxymugineic-acid 2'-dioxygenase [Triticum urartu] | + | + | - | 15.3 | up | 4.3 | up | 1.3 | up |
| 77 | Ta.23681.1.S1_a_at | Ta.23681 | Hypothetical protein F775_24401 [Aegilops tauschii] | + | + | - | 13.9 | up | 8.6 | up | 19.3 | up |
| 78 | Ta.9809.2.S1_at | --- | Beta-fructofuranosidase, insoluble isoenzyme 4 [Aegilops tauschii] | + | + | - | 12.6 | up | 1.7 | up | 1.1 | up |
| 79 | Ta.28744.1.S1_at | --- | Hypothetical protein F775_23873 [Aegilops tauschii] | + | + | - | 11.3 | up | 11 | up | 35.2 | up |
| 80 | Ta.1622.2.S1_at | Ta.1622 | Sugar transporter ERD6-like protein 5 [Aegilops tauschii] | + | + | - | 10 | down | 2.4 | down | 1.4 | down |
| 81 | Ta.23366.2.S1_at | Ta.54251 | Peroxidase 66 [Triticum urartu] | + | + | - | 2.6 | up | 10 | up | 60 | up |
| 82 | Ta.14489.1.S1_at | Ta.13380 | pTACR7 [Triticum aestivum] | + | + | - | 2.4 | down | 7.6 | down | 29 | down |
| 83 | Ta.23366.2.S1_x_at | Ta.54251 | Peroxidase 66 [Triticum urartu] | + | + | - | 2.4 | up | 5.6 | up | 28.9 | up |
| 84 | Ta.3527.1.S1_at | Ta.41090 | Hypothetical protein TRIUR3_34067 [Triticum urartu] | + | + | - | 6.2 | up | 8.1 | up | 28.3 | up |
| 85 | Ta.5129.2.A1_a_at | Ta.5129 | Heat stress transcription factor A-9 [Aegilops tauschii] | + | + | - | 7.5 | down | 5.4 | down | 25.1 | down |
| 86 | Ta.5243.2.S1_a_at | Ta.5243 | Hypothetical protein TRIUR3_06223 [Triticum urartu] | + | + | - | 2.3 | down | 4.9 | down | 19 | down |
| 87 | Ta.21106.1.A1_at | --- | Hypothetical protein TRIUR3_30277 [Triticum urartu] | + | + | - | 4.9 | up | 5.7 | up | 16.2 | up |
| 88 | TaAffx.5865.2.A1_at | --- | Hypothetical protein TRIUR3_27593 [Triticum urartu] | + | + | - | 1.4 | down | 2.9 | up | 14.2 | up |
| 89 | TaAffx.30606.1.S1_at | --- | Katanin p80 WD40 repeat-containing subunit B1-like protein 1 [Triticum urartu] | + | + | - | 1.2 | down | 2.3 | up | 14 | up |
| 90 | TaAffx.31445.1.S1_at | --- | No significant similarity found | + | + | - | 1.4 | down | 3.6 | up | 11.1 | up |
| 91 | TaAffx.82110.1.S1_at | --- | Hypothetical protein TRIUR3_18607 [Triticum urartu] | + | + | - | 1.4 | down | 2.5 | up | 10.5 | up |
| 92 | TaAffx.86295.1.S1_at | --- | zf-MYND domain-containing protein [Triticum aestivum] | + | + | - | 1.3 | down | 2.1 | up | 10.4 | up |
| 93 | Ta.12328.2.A1_at | Ta.12328 | Hypothetical protein F775_32343 [Aegilops tauschii] | + | + | - | 1.1 | down | 2.3 | up | 10.1 | up |
| 94 | Ta.2415.2.S1_a_at | --- | gliadin/avenin-like seed protein [Triticum aestivum] | + | - | - | 17.4 | up | 2.1 | up | 1.1 | down |
| 95 | Ta.14507.2.S1_at | Ta.54186 | Cytosolic Fe-S cluster assembly factor NUBP1-like protein [Triticum urartu] | + | - | - | 29.9 | down | 3.6 | down | 7.6 | down |
| 96 | Ta.11896.1.A1_s_at | Ta.11896 | Hypothetical protein TRIUR3_24659 [Triticum urartu] | + | - | - | 24.9 | up | 7.9 | up | 3.7 | up |
| 97 | Ta.14446.1.A1_at | Ta.54013 | Hypothetical protein TRIUR3_05569 [Triticum urartu] | + | - | - | 14.8 | down | 13.2 | down | 9.9 | down |
| 98 | Ta.19222.1.S1_x_at | Ta.13926 | No significant similarity found | + | - | - | 14.1 | up | 3.9 | up | 3.2 | up |
| 99 | Ta.7509.3.S1_at | Ta.65964 | Hypothetical protein F775_05928 [Aegilops tauschii] | + | - | - | 14 | down | 26.9 | down | 22 | down |
| 100 | Ta.2434.3.A1_at | Ta.2434 | S-formylglutathione hydrolase [Aegilops tauschii] | + | - | - | 13.7 | down | 11.9 | down | 9.6 | down |
| 101 | Ta.8883.2.S1_at | Ta.8883 | Hypothetical protein F775_27368 [Aegilops tauschii] | + | - | - | 12 | down | 7.9 | down | 9.2 | down |
| 102 | TaAffx.78552.1.S1_x_at | Ta.55330 | Hypothetical protein TRIUR3_27901 [Triticum urartu] | + | - | - | 11.7 | up | 1.7 | up | 2.9 | up |
| 103 | Ta.24218.1.S1_at | Ta.24218 | Hypothetical protein TRIUR3_02605 [Triticum urartu] | + | - | - | 11.6 | up | 6.9 | up | 3.6 | up |
| 104 | Ta.7430.1.S1_at | --- | GATA transcription factor 17 [Triticum urartu] | + | - | - | 11.4 | up | 5.3 | up | 6.4 | up |
| 105 | Ta.23352.1.S1_at | --- | Putative serine/threonine-protein kinase-like protein CCR3 [Aegilops tauschii] | + | - | - | 10.8 | up | 5.8 | up | 6.1 | up |
| 106 | Ta.5278.1.S1_at | Ta.5278 | Hypothetical protein TRIUR3_04715 [Triticum urartu] | + | - | - | 10.1 | down | 4.2 | down | 3 | down |
| 107 | Ta.27445.1.S1_at | Ta.27445 | Cell division protease ftsH-like protein, chloroplastic [Aegilops tauschii] | + | - | - | 9.2 | down | 13.4 | down | 28.6 | down |
| 108 | Ta.14050.1.S1_at | Ta.14050 | Hypothetical protein F775_27373 [Aegilops tauschii] | + | - | - | 2.8 | up | 3.7 | up | 37.3 | up |
| 109 | TaAffx.91902.1.A1_at | Ta.47695 | No significant similarity found | + | - | - | 4.2 | up | 5.5 | up | 32.6 | up |
| 110 | Ta.28759.1.A1_at | Ta.28759 | Hypothetical protein F775_09062 [Aegilops tauschii] | + | - | - | 5.7 | up | 2.7 | up | 14.7 | up |
| 111 | Ta.28368.2.S1_at | Ta.46258 | type 1 non specific lipid transfer protein precursor [Triticum aestivum] | - | + | + | 11.9 | down | 1.1 | up | 1 | down |
| 112 | Ta.24969.1.S1_s_at | Ta.24969 | low-molecular-weight glutenin subunit [Triticum aestivum] | - | + | + | 113.7 | up | 1.1 | down | 1.3 | down |
| 113 | Ta.23142.10.S1_x_at | Ta.56908 | gamma-gliadin/LMW-glutenin chimera Ch5 precursor [Triticum aestivum] | - | + | + | 46.4 | up | 1 | down | 1.1 | up |
| 114 | Ta.23142.8.S1_x_at | Ta.24969 | LMW-glutenin subunit [Triticum aestivum] | - | + | + | 39.6 | up | 1 | down | 1 | up |
| 115 | Ta.22828.2.S1_at | Ta.50492 | Putative lipoxygenase 3 [Triticum urartu] | - | + | + | 12.7 | down | 1.9 | up | 1.4 | up |
| 116 | Ta.2448.1.S1_at | --- | avenin-like protein [Triticum aestivum] | - | + | + | 77.9 | up | 1 | down | 1.3 | down |
| 117 | Ta.24114.14.S1_x_at | Ta.55042 | gamma-gliadin [Triticum aestivum] | - | + | + | 102.3 | up | 1.1 | up | 1.1 | down |
| 118 | Ta.30782.9.S1_x_at | Ta.54261 | gamma-gliadin [Triticum aestivum] | - | + | + | 44.4 | up | 1.1 | up | 1.9 | down |
| 119 | Ta.7756.2.S1_a_at | Ta.7756 | Bowman-Birk trypsin inhibitor-like protein | - | + | + | 27.8 | up | 1.6 | down | 1.2 | down |
| 120 | Ta.7756.2.S1_x_at | Ta.7756 | Bowman-Birk trypsin inhibitor-like protein | - | + | + | 25.5 | up | 1.5 | down | 1.7 | down |
| 121 | Ta.14503.1.S1_at | Ta.67690 | Cys peroxiredoxin PER1 | - | + | + | 11 | up | 1.3 | down | 1.3 | up |
| 122 | Ta.14625.2.S1_x_at | Ta.14625 | low molecular weight glutenin subunit [Triticum aestivum] | - | + | + | 70.3 | up | 1 | down | 1.1 | down |
| 123 | Ta.23142.7.S1_x_at | Ta.67635 | gamma gliadin [Triticum aestivum] | - | + | + | 49.3 | up | 1 | down | 1 | down |
| 124 | Ta.1257.2.A1_x_at | Ta.50490 | truncated HMW-glutenin subunit 1By9 [Triticum aestivum subsp. yunnanense] | - | + | + | 35.6 | up | 1.3 | down | 1.3 | up |
| 125 | Ta.14039.1.S1_x_at | Ta.14039 | Hypothetical protein F775_05051 [Aegilops tauschii] | - | + | + | 23.5 | up | 1.4 | down | 1.1 | down |
| 126 | Ta.21488.1.A1_x_at | Ta.21488 | No significant similarity found | - | + | + | 15.7 | down | 18.2 | up | 9.1 | up |
| 127 | TaAffx.128418.15.S1_s_at | Ta.58931 | No significant similarity found | - | + | + | 14 | down | 1.4 | up | 3.6 | up |
| 128 | TaAffx.24287.1.S1_s_at | --- | Hypothetical protein F775_28674 [Aegilops tauschii] | - | + | + | 13.5 | up | 1.1 | down | 1.1 | up |
| 129 | Ta.27768.1.S1_at | Ta.27768 | Hypothetical protein F775_28945 [Aegilops tauschii] | - | + | + | 13.4 | down | 1.3 | up | 1.1 | up |
| 130 | TaAffx.24287.1.S1_at | --- | Hypothetical protein F775_28674 [Aegilops tauschii] | - | + | + | 12.8 | up | 1.1 | down | 1.2 | up |
| 131 | TaAffx.4544.2.S1_s_at | --- | cytochrome c heme attachment protein (chloroplast) [Aegilops speltoides] | - | + | + | 11.8 | down | 1.7 | up | 3.8 | up |
| 132 | Ta.24550.2.S1_x_at | --- | Hypothetical protein TRIUR3_13108 [Triticum urartu] | - | + | + | 11.2 | up | 1.5 | down | 1.1 | down |
| 133 | TaAffx.132743.10.S1_at | Ta.56634 | ent-copalyl diphosphate synthase [Triticum aestivum] | - | + | + | 11.1 | down | 3.3 | up | 2.6 | up |
| 134 | TaAffx.38359.1.S1_at | --- | Non-specific lipid-transfer protein 2G [Aegilops tauschii] | - | + | + | 11.1 | up | 1.4 | down | 1.3 | down |
| 135 | Ta.27107.1.S1_at | Ta.48778 | Glucan endo-1,3-beta-glucosidase GIV [Aegilops tauschii] | - | + | + | 10.7 | up | 1.1 | down | 1.1 | up |
| 136 | Ta.20518.1.S1_at | Ta.46137 | Endogenous alpha-amylase/subtilisin inhibitor | - | + | + | 10.3 | up | 1 | up | 1 | down |
| 137 | Ta.16059.1.S1_x_at | Ta.16059 | chitinase II precursor [Triticum aestivum] | - | + | + | 10.2 | down | 1.1 | up | 1.1 | up |
| 138 | Ta.13400.2.A1_at | Ta.13400 | tonoplast intrinsic protein 3;1 [Triticum aestivum] | - | + | + | 10.2 | up | 1.1 | up | 1 | down |
| 139 | TaAffx.74416.2.S1_at | - | Putative protein phosphatase 2C 64 [Triticum urartu] | - | + | + | 2.9 | down | 10.8 | up | 8.4 | up |
| 140 | Ta.19.1.S1_at | Ta.19 | type V Thionin [Aegilops tauschii] | - | + | - | 18.3 | up | 1.2 | down | 1.1 | down |
| 141 | Ta.1314.1.S1_s_at | Ta.118 | Serpin-Z1B [Aegilops tauschii] | - | + | - | 14.7 | up | 1 | down | 1.5 | down |
| 142 | Ta.14614.1.S1_at | Ta.63439 | putative puroindoline-like protein [Triticum aestivum] | - | + | - | 10.1 | up | 1 | down | 1.4 | up |
| 143 | Ta.115.1.S1_at | Ta.115 | puroindoline b protein [Triticum aestivum] | - | + | - | 34.8 | up | 1.2 | down | 1.1 | up |
| 144 | Ta.23141.1.S1_at | Ta.41965 | puroindoline a [Triticum aestivum] | - | + | - | 1.3 | down | 1.8 | down | 22.2 | up |
| 145 | Ta.28466.1.S1_at | Ta.56896 | PUP88 protein; member of trypsin/a-amylase inhibitors family from cereals [Triticum aestivum] | - | + | - | 11.6 | up | 1.7 | down | 1.2 | down |
| 146 | Ta.817.1.S1_a_at | Ta.817 | Alpha-amylase/trypsin inhibitor CM3 | - | + | - | 24 | up | 1.1 | down | 1.7 | up |
| 147 | Ta.2446.2.S1_at | Ta.56892 | alpha amylase inhibitor CM1, partial [Triticum aestivum] | - | + | - | 32.2 | up | 1.1 | down | 1.3 | down |
| 148 | Ta.6699.1.S1_at | Ta.28482 | alpha-gliadin [Triticum aestivum] | - | + | - | 32.5 | up | 1.1 | down | 1.3 | down |
| 149 | Ta.10140.1.S1_at | Ta.10140 | peroxidase 1 [Triticum aestivum] | - | + | - | 17 | up | 1 | up | 1.1 | down |
| 150 | Ta.1315.1.S1_at | Ta.1315 | monomeric alpha-amylase inhibitor [Triticum aestivum] | - | + | - | 14.9 | up | 1.2 | down | 1 | down |
| 151 | Ta.58.1.S1_at | Ta.56181 | Alpha-amylase/trypsin inhibitor CM16 | - | + | - | 26.3 | up | 1.1 | down | 1.2 | up |
| 152 | Ta.23142.1.S1_x_at | Ta.56908 | LMW-glutenin [Triticum aestivum] | - | + | - | 23.7 | up | 1.1 | down | 1.2 | down |
| 153 | Ta.30756.1.S1_x_at | Ta.48416 | LMW-glutenin subunit group 3 type II [Triticum aestivum] | - | + | - | 48.3 | up | 1.1 | up | 1.5 | up |
| 154 | Ta.23142.6.S1_x_at | Ta.39587 | LMW-glutenin subunit B3-2 [Triticum aestivum] | - | + | - | 46.9 | up | 1 | down | 1.2 | down |
| 155 | Ta.13254.1.S1_x_at | Ta.48405 | high molecular weight glutenin y-type, partial [Triticum aestivum] | - | + | - | 17.6 | up | 2.4 | down | 1.6 | up |
| 156 | Ta.2415.1.S1_at | --- | gliadin/avenin-like seed protein [Triticum aestivum] | - | + | - | 35.1 | up | 1.6 | up | 1.4 | up |
| 157 | Ta.2415.3.S1_x_at | --- | gliadin/avenin-like seed protein [Triticum aestivum] | - | + | - | 31.6 | up | 1.5 | up | 1.3 | down |
| 158 | TaAffx.128418.2.S1_x_at | Ta.66066 | gamma-gliadin [Triticum aestivum] | - | + | - | 99.6 | up | 1.1 | up | 2.5 | up |
| 159 | Ta.23798.1.S1_s_at | Ta.23798 | CM 17 protein precursor [Triticum aestivum] | - | + | - | 25.7 | up | 1.1 | down | 1.3 | up |
| 160 | Ta.23798.1.S1_at | Ta.23798 | CM 17 protein precursor [Triticum aestivum] | - | + | - | 32.6 | up | 1.1 | down | 1.2 | up |
| 161 | Ta.1308.1.S1_at | Ta.1308 | beta purothionin [Triticum urartu] | - | + | - | 30.9 | up | 1.2 | down | 1.2 | down |
| 162 | Ta.1308.1.S1_x_at | Ta.1308 | beta purothionin [Triticum urartu] | - | + | - | 27.6 | up | 1.1 | down | 1.1 | up |
| 163 | TaAffx.42864.3.S1_x_at | Ta.54476 | Avenin-like a6; | - | + | - | 102.1 | up | 1.2 | up | 1.1 | down |
| 164 | Ta.905.2.S1_at | Ta.54476 | Avenin-like a1 | - | + | - | 45.1 | up | 1.1 | up | 1.1 | up |
| 165 | Ta.23141.2.S1_at | Ta.28296 | dimeric alpha-amylase inhibitor [Triticum aestivum] | - | + | - | 42.8 | up | 1 | down | 1.3 | down |
| 166 | Ta.23967.1.S1_x_at | Ta.23967 | alpha purothionin [Triticum aestivum] | - | + | - | 21.6 | up | 1.9 | down | 1.7 | down |
| 167 | Ta.23967.1.S1_s_at | Ta.23967 | alpha purothionin [Triticum aestivum] | - | + | - | 20 | up | 1.2 | down | 1.1 | down |
| 168 | Ta.91.1.S1_at | Ta.91 | alpha-1 purothionin [Triticum aestivum] | - | + | - | 15.6 | up | 1.1 | down | 1.2 | down |
| 169 | Ta.24114.11.S1_x_at | Ta.24085 | alpha/beta-gliadin [Triticum aestivum] | - | + | - | 56 | up | 1.2 | down | 1.1 | up |
| 170 | Ta.24114.15.A1_x_at | Ta.24085 | alpha-gliadin [Triticum aestivum] | - | + | - | 33 | up | 1 | down | 1.4 | down |
| 171 | Ta.27778.6.S1_x_at | Ta.24085 | alpha-gliadin [Triticum aestivum] | - | + | - | 10.2 | up | 1.1 | down | 1.4 | up |
| 172 | Ta.2446.1.S1_a_at | --- | Alpha-amylase/trypsin inhibitor CM2 | - | + | - | 34.2 | up | 1 | down | 1.1 | up |
| 173 | Ta.9233.2.S1_a_at | Ta.35848 | 27k protein [Triticum aestivum] | - | + | - | 27.9 | up | 1.1 | up | 1 | down |
| 174 | Ta.9233.1.S1_at | Ta.35848 | 27k protein [Triticum aestivum] | - | + | - | 17 | up | 1 | up | 1.3 | up |
| 175 | Ta.9402.1.S1_at | Ta.9402 | 19 kDa globulin [Triticum aestivum] | - | + | - | 18.3 | up | 1.1 | up | 1.2 | down |
| 176 | Ta.9402.1.S1_x_at | Ta.9402 | 19 kDa globulin [Triticum aestivum] | - | + | - | 18.1 | up | 1 | up | 1.3 | down |
| 177 | Ta.69.2.S1_x_at | Ta.66027 | 15kDa grain softness protein [Triticum aestivum] | - | + | - | 42.8 | up | 1 | up | 1 | down |
| 178 | Ta.840.1.S1_at | Ta.840 | 15kDa grain softness protein [Triticum aestivum] | - | + | - | 30.5 | up | 1.3 | down | 1.6 | down |
| 179 | Ta.22918.1.S1_x_at | Ta.28542 | Hypothetical protein F775_43830 [Aegilops tauschii] | - | + | - | 40.4 | up | 1.1 | up | 1.9 | up |
| 180 | Ta.2446.1.S1_x_at | Ta.54224 | putative alpha-amylase inhibitor CM2, partial [Triticum aestivum] | - | + | - | 40.1 | up | 1.1 | down | 1 | up |
| 181 | Ta.23141.3.S1_x_at | Ta.28296 | dimeric alpha-amylase inhibitor [Triticum aestivum] | - | + | - | 39.4 | up | 1 | down | 1.2 | down |
| 182 | TaAffx.52381.1.S1_at | --- | triticin [Triticum aestivum] | - | + | - | 36.7 | up | 1.4 | up | 1.1 | down |
| 183 | Ta.242.2.S1_at | Ta.55081 | Hypothetical protein TRIUR3_24082 [Triticum urartu] | - | + | - | 34.6 | up | 1.7 | up | 1 | down |
| 184 | Ta.2446.1.S1_at | Ta.54224 | Alpha-amylase/trypsin inhibitor CM2 | - | + | - | 34.6 | up | 1 | up | 1.4 | up |
| 185 | Ta.1550.1.S1_at | Ta.55497 | trypsin inhibitor CMx precursor - wheat | - | + | - | 30.9 | up | 1.1 | down | 1.4 | up |
| 186 | Ta.25057.1.S1_at | Ta.56702 | No significant similarity found | - | + | - | 28.4 | up | 1.2 | down | 2.4 | down |
| 187 | Ta.1549.1.S1_at | --- | Aspartic proteinase oryzasin-1 [Triticum urartu] | - | + | - | 22.8 | up | 1 | up | 1.1 | up |
| 188 | TaAffx.51309.1.S1_at | --- | 60S ribosomal protein L4-1 [Triticum urartu] | - | + | - | 21.7 | down | 1.2 | up | 1.2 | up |
| 189 | Ta.1480.2.S1_a_at | Ta.1480 | Hypothetical protein F775_25878 [Aegilops tauschii] | - | + | - | 21.3 | up | 1.4 | down | 1.8 | down |
| 190 | Ta.6175.2.A1_x_at | Ta.35463 | gamma-gliadin [Triticum aestivum] | - | + | - | 21.2 | up | 1.9 | up | 2 | down |
| 191 | Ta.2415.2.S1_x_at | Ta.2415 | gliadin/avenin-like seed protein [Triticum aestivum] | - | + | - | 20.5 | up | 1.5 | up | 1.1 | down |
| 192 | Ta.1480.2.S1_x_at | Ta.1480 | Hypothetical protein F775_25878 [Aegilops tauschii] | - | + | - | 20.1 | up | 1.4 | down | 1.8 | down |
| 193 | TaAffx.120246.1.S1_x_at | Ta.50490 | glutenin high molecular weight subunit [Triticum aestivum] | - | + | - | 19.5 | up | 1.3 | down | 1.4 | down |
| 194 | TaAffx.4219.1.S1_at | Ta.9963 | Hypothetical protein F775_26803 [Aegilops tauschii] | - | + | - | 18.9 | up | 1.1 | down | 1.3 | up |
| 195 | Ta.27386.1.S1_x_at | --- | HMW-glutenin subunit Ax2* [Triticum aestivum] | - | + | - | 18.5 | up | 3.8 | down | 5 | down |
| 196 | Ta.9940.1.A1_at | --- | gamma-gliadin [Triticum aestivum] | - | + | - | 18.1 | up | 1.1 | up | 3.1 | down |
| 197 | Ta.18957.1.S1_at | --- | Hypothetical protein F775_02836 [Aegilops tauschii] | - | + | - | 17.3 | up | 2.5 | up | 1 | up |
| 198 | Ta.556.2.A1_at | Ta.55487 | Hypothetical protein TRIUR3_35361 [Triticum urartu] | - | + | - | 16.5 | down | 1 | up | 1 | down |
| 199 | Ta.1480.1.S1_x_at | Ta.1480 | Hypothetical protein F775_25878 [Aegilops tauschii] | - | + | - | 16.4 | up | 1.3 | down | 1.2 | up |
| 200 | Ta.18957.1.S1_x_at | --- | Hypothetical protein F775_02836 [Aegilops tauschii] | - | + | - | 16.1 | up | 2.2 | up | 1 | up |
| 201 | Ta.1545.1.S1_at | Ta.54173 | Stem 28 kDa glycoprotein [Triticum urartu] | - | + | - | 14.7 | up | 1.2 | up | 1.1 | up |
| 202 | Ta.14111.1.S1_at | Ta.14111 | Hypothetical protein TRIUR3_06942 [Triticum urartu] | - | + | - | 14.4 | up | 1.4 | down | 1.2 | up |
| 203 | Ta.1411.1.S1_s_at | Ta.1411 | trypsin inhibitor [Triticum monococcum subsp. monococcum] | - | + | - | 14.3 | up | 1.9 | down | 1.2 | down |
| 204 | Ta.14039.2.S1_x_at | Ta.14039 | Hypothetical protein TRIUR3_01199 [Triticum urartu] | - | + | - | 14.3 | up | 1.4 | down | 1 | up |
| 205 | Ta.14111.1.S1_x_at | Ta.14111 | Hypothetical protein TRIUR3_06942 [Triticum urartu] | - | + | - | 13.8 | up | 1.3 | down | 1.3 | up |
| 206 | Ta.22864.1.S1_a_at | Ta.54668 | Hypothetical protein F775_27244 [Aegilops tauschii] | - | + | - | 13.5 | up | 2.3 | down | 2.5 | down |
| 207 | Ta.9999.1.S1_at | Ta.9999 | serpin 2 [Triticum aestivum] | - | + | - | 13 | up | 1.1 | down | 1.7 | down |
| 208 | Ta.9884.1.S1_at | --- | Hypothetical protein F775_30947 [Aegilops tauschii] | - | + | - | 12.8 | up | 1.4 | down | 1.2 | down |
| 209 | Ta.22925.1.S1_at | Ta.67730 | Bowman-Birk type trypsin inhibitor [Triticum urartu] | - | + | - | 12.5 | up | 2.2 | up | 1.2 | down |
| 210 | Ta.9990.1.S1_at | Ta.9990 | Endoglucanase 11 [Triticum urartu] | - | + | - | 12.5 | up | 1.1 | up | 1 | up |
| 211 | Ta.154.5.S1_x_at | Ta.54396 | Globulin-1 S allele [Triticum urartu] | - | + | - | 12.2 | up | 1.1 | down | 1 | down |
| 212 | Ta.9884.1.S1_x_at | --- | Hypothetical protein F775_30947 [Aegilops tauschii] | - | + | - | 12.1 | up | 1.4 | down | 1.3 | down |
| 213 | Ta.9990.1.S1_x_at | Ta.9990 | Endoglucanase 11 [Triticum urartu] | - | + | - | 12.1 | up | 1.1 | up | 1.2 | up |
| 214 | Ta.10826.1.A1_a_at | Ta.58141 | Protein CUP-SHAPED COTYLEDON 2 [Aegilops tauschii] | - | + | - | 12 | up | 1 | down | 1.5 | up |
| 215 | Ta.20517.1.S1_a_at | Ta.20517 | Hypothetical protein F775_52412 [Aegilops tauschii] | - | + | - | 11.7 | up | 1.1 | down | 1.1 | up |
| 216 | TaAffx.144000.1.S1_x_at | --- | Hypothetical protein TRIUR3_19989 [Triticum urartu] | - | + | - | 11.5 | down | 1.7 | down | 1.3 | down |
| 217 | Ta.20517.3.S1_x_at | Ta.20517 | Hypothetical protein TRIUR3_22438 [Triticum urartu] | - | + | - | 10.9 | up | 1.1 | down | 1.1 | up |
| 218 | Ta.10147.2.S1_x_at | Ta.10147 | Hypothetical protein F775_30642 [Aegilops tauschii] | - | + | - | 10.6 | up | 1 | down | 1.3 | up |
| 219 | Ta.792.1.S1_at | --- | Aspartic proteinase nepenthesin-2 [Triticum urartu] | - | + | - | 10.5 | up | 1.2 | down | 1.1 | up |
| 220 | Ta.22038.1.S1_at | Ta.22038 | Non-specific lipid-transfer protein 2G [Aegilops tauschii] | - | + | - | 10.4 | up | 1.8 | down | 1.3 | down |
| 221 | Ta.10018.1.S1_at | --- | Hypothetical protein F775_27244 [Aegilops tauschii] | - | + | - | 10.4 | up | 1.5 | down | 2.2 | up |
| 222 | Ta.21933.1.S1_a_at | Ta.21933 | putative oleosin [Triticum aestivum] | - | + | - | 10.3 | up | 1.1 | down | 1.1 | down |
| 223 | Ta.25258.1.S1_at | Ta.57891 | Protein CUP-SHAPED COTYLEDON 2 [Aegilops tauschii] | - | + | - | 10.2 | up | 1.3 | down | 1.7 | up |
| 224 | Ta.885.1.S1_at | Ta.885 | Protein SRG1 [Aegilops tauschii] | - | + | - | 10.2 | up | 1.2 | down | 1.2 | up |
| 225 | Ta.24547.1.S1_at | Ta.56021 | Hypothetical protein TRIUR3_13855 [Triticum urartu] | - | + | - | 10.1 | up | 1.1 | up | 2.2 | up |
| 226 | Ta.9809.1.A1_at | Ta.9809 | Beta-fructofuranosidase, insoluble isoenzyme 4 [Aegilops tauschii] | - | + | - | 10.1 | up | 1.1 | up | 1.4 | down |
| 227 | Ta.10826.2.A1_x_at | Ta.58141 | NAC transcription factor NAM [Triticum aestivum] | - | + | - | 10 | up | 1 | down | 1.4 | up |
| 228 | Ta.6210.1.S1_at | Ta.9520 | Hypothetical protein TRIUR3_17216 [Triticum urartu] | - | + | - | 1.2 | up | 1.2 | up | 18.6 | up |
| 229 | TaAffx.25602.1.S1_s_at | --- | Hypothetical protein TRIUR3_07329 [Triticum urartu] | - | + | - | 1.3 | down | 5.3 | up | 16.2 | up |
| 230 | TaAffx.109268.1.S1_at | --- | TaCBF11 [Triticum aestivum] | - | + | - | 1.4 | down | 4.6 | up | 13.9 | up |
| 231 | Ta.20949.1.A1_at | --- | Lactoylglutathione lyase [Triticum urartu] | - | + | - | 1.2 | up | 1 | down | 12.7 | down |
| 232 | Ta.5847.1.S1_x_at | Ta.5847 | Universal stress protein A-like protein [Triticum urartu] | - | + | - | 2 | down | 5.6 | up | 12.4 | up |
| 233 | Ta.5847.1.S1_at | Ta.5847 | Universal stress protein A-like protein [Triticum urartu] | - | + | - | 2.2 | down | 5.7 | up | 11.7 | up |
| 234 | TaAffx.110627.1.S1_at | --- | No significant similarity found | - | + | - | 1.3 | down | 2.8 | up | 10.5 | up |
| 235 | TaAffx.65642.1.S1_at | --- | No significant similarity found | - | + | - | 1.4 | down | 2.8 | up | 10.3 | up |
| 236 | TaAffx.131916.5.S1_at | --- | Hypothetical protein TRIUR3_11624 [Triticum urartu] | - | + | - | 1.1 | up | 3.4 | up | 10.2 | up |
