# Supplementary figures and images for: Genome-wide transcriptome study in wheat identified candidate genes related to processing quality, majority of them showing interaction (quality x development) and having temporal and spatial distributions
Source: BMC Genomics. 2014 Jan 16;15:29. doi: 10.1186/1471-2164-15-29 (PMC3897974; doi:10.1186/1471-2164-15-29)

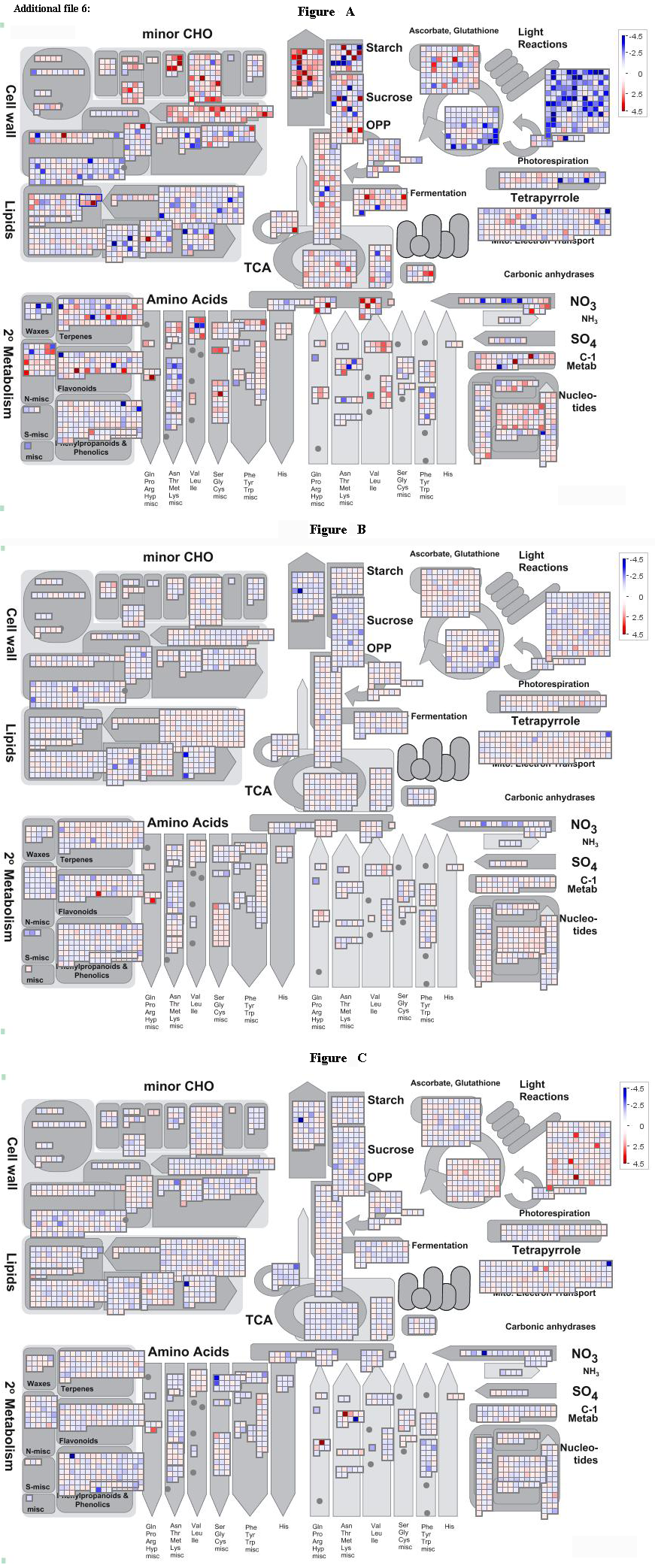

Supplement: Additional file 6 — Display of genes of 35,472 probe sets to metabolic pathways using MapMan software (version 3.5.1R2)[43-45]. The transcript levels of genes in several metabolic pathways were visualized by colour scales (+4.5 red to blue –4.5), where red and blue represents an increase and decrease of expression (log2 of fold change value), respectively in good quality varieties in comparison to poor making varieties in 7 days after anthesis (Top figure A), 14 DAA (Middle figure B), and 28 DAA (Last figure C). [file 1471-2164-15-29-S6.tiff]
