# Supplementary material for: Genome-wide transcriptome study in wheat identified candidate genes related to processing quality, majority of them showing interaction (quality x development) and having temporal and spatial distributions
Source: BMC Genomics. 2014 Jan 16;15:29. doi: 10.1186/1471-2164-15-29 (PMC3897974; doi:10.1186/1471-2164-15-29)
Supplement: Additional file 7 — Detail of the four India wheat varieties used in transcriptome studies. [file 1471-2164-15-29-S7.docx]

**Additional file 7:** Detail of the four India wheat varieties used in transcriptome studies

| **Variety** | **Parentage** | **Year (releasing committee)** | **Developed by** | **Area of adoption** | **Source** | **Yield potential** (**Q/ha)** |
| --- | --- | --- | --- | --- | --- | --- |
| C306 | RGN/CSK3//2*C591/3/C217/N14//C281) | 1965 (CVRC) | HISER | NWPZ/NEPZ | MPUAT, Kota (RJ) | 36.0 |
| LOK1 | S308/S331 | 1981 (CVRC) | SANOSARA | CZ | JNKVV, Jabalpur (MP) | 45.4 |
| Sonalika | II54.388/AN/3/YT54/N 10B/LR 64 | 1969 (CVRC) | N.DELHI | NW/NWPZ | DWR, Karnal (HR) | 45.5 |
| WH291 | HD1925/HD832 //23584 | 1985 (CVRC) | HISAR | NWPZ | DWR, Karnal (HR) | 44.0 |

CVRC : Central Varietal Release Committee

NWPZ : North Western Plains Zone

NEPZ : North Eastern Plains Zone

CZ : Central zone

NW : Northwest

MPUAT : Maharana Pratap University of Agriculture and Technology

JNKVV : Jawaharlal Nehru Krishi Vishwavidyalaya

DWR : Directorate of Wheat Research
