# Supplementary material for: Genome-wide transcriptome study in wheat identified candidate genes related to processing quality, majority of them showing interaction (quality x development) and having temporal and spatial distributions
Source: BMC Genomics. 2014 Jan 16;15:29. doi: 10.1186/1471-2164-15-29 (PMC3897974; doi:10.1186/1471-2164-15-29)
Supplement: Additional file 8 — Quality evaluations of the extracted RNAs from three replications of each of the four Indian wheat varieties (C306, Lok1, Sonalika, and WH291) at three seed development stages [7, 14, and 28 days after anthesis (DAA)]. [file 1471-2164-15-29-S8.docx]

**Additional file 8:** Quality evaluations of the extracted RNAs from three replications of each of four Indian wheat varieties (C306, Lok1, Sonalika, and WH291) at three seed development stages [7, 14, and 28 days after anthesis (DAA)] on three different systems

| **Varieties_seed developing stage_replication number** | **RNA concentration (ng/µl)**  **(Tecan’s NanoQuant)** | **RNA Integrity Number (RIN)**  **(Agilent’s Bioanalyzer)** | **% P hybridization**  **(Affymetrix’s microarray scanner)** |
| --- | --- | --- | --- |
| C306_7DAA_Rep # 1 | 2799 | 10.0 | 37.75983 |
| C306_7DAA_Rep # 2 | 2582 | 8.7 | 36.299561 |
| C306_7DAA_Rep # 3 | 2167 | 8.4 | 38.370045 |
| C306_14DAA_Rep # 1 | 1867 | 8.7 | 35.571873 |
| C306_14DAA_Rep # 2 | 1945 | 7.9 | 38.472836 |
| C306_14DAA_Rep # 3 | 1345 | 7.9 | 36.159245 |
| C306_28DAA_Rep # 1 | 1151 | 8.3 | 33.424702 |
| C306_28DAA_Rep # 2 | 1567 | 7.8 | 35.935715 |
| C306_28DAA_Rep # 3 | 1532 | 7.6 | 37.958885 |
| LOK1_7DAA_Rep # 1 | 1693 | 8.2 | 34.932289 |
| LOK1_7DAA_Rep # 2 | 1933 | 7.8 | 34.532551 |
| LOK1_7DAA_Rep # 3 | 1290 | 7.9 | 38.658836 |
| LOK1_14DAA_Rep # 1 | 1678 | 7.9 | 36.154346 |
| LOK1_14DAA_Rep # 2 | 1234 | 7.9 | 35.519661 |
| LOK1_14DAA_Rep # 3 | 1456 | 7.8 | 37.557514 |
| LOK1_28DAA_Rep # 1 | 1435 | 7.3 | 33.772232 |
| LOK1_28DAA_Rep # 2 | 989 | 7.2 | 32.054169 |
| LOK1_28DAA_Rep # 3 | 1011 | 7.1 | 34.431393 |
| Sonalika_7DAA_Rep # 1 | 1675 | 9.1 | 36.54593 |
| Sonalika_7DAA_Rep # 2 | 2711 | 8.6 | 38.38147 |
| Sonalika_7DAA_Rep # 3 | 2151 | 8.0 | 37.68315 |
| Sonalika_14DAA_Rep # 1 | 1096 | 8.6 | 36.17719 |
| Sonalika_14DAA_Rep # 2 | 1123 | 7.9 | 36.8233 |
| Sonalika_14DAA_Rep # 3 | 1345 | 7.6 | 37.8512 |
| Sonalika_28DAA_Rep # 1 | 1348 | 7.9 | 37.86752 |
| Sonalika_28DAA_Rep # 2 | 1674 | 7.8 | 36.39419 |
| Sonalika_28DAA_Rep # 3 | 1823 | 7.6 | 36.42682 |
| WH291_7DAA_Rep # 1 | 1816 | 8.7 | 33.70371 |
| WH291_7DAA_Rep # 2 | 908 | 7.7 | 36.35993 |
| WH291_7DAA_Rep # 3 | 1619 | 8.4 | 37.40578 |
| WH291_14DAA_Rep # 1 | 2693 | 8.0 | 35.50008 |
| WH291_14DAA_Rep # 2 | 1952 | 7.7 | 35.32877 |
| WH291_14DAA_Rep # 3 | 1595 | 7.5 | 37.20183 |
| WH291_28DAA_Rep # 1 | 2021 | 7.9 | 35.66324 |
| WH291_28DAA_Rep # 2 | 1999 | 7.5 | 35.27492 |
| WH291_28DAA_Rep # 3 | 1789 | 7.4 | 34.77239 |

**Gel images showing RNA Integrity Number (RIN) of the extracted RNAs on Agilent’s 2100 Bioanalyzer**

|  | **7DAA** | **14DAA** | **28DAA** |
| --- | --- | --- | --- |
| **C306** |   RIN: 10 |   RIN: 8.7 |   RIN: 8.3 |
| **Lok1** |   RIN: 8.2 |   RIN: 7.9 |   RIN: 7.3 |
| **Sonalika** |   RIN: 9.1 |   RIN: 8.6 |   RIN: 7.9 |
| **WH291** |   RIN: 8.7 |   RIN: 8.0 |   RIN: 7.9 |
